# Multiple genetic lineages challenge the monospecific status of the West African endemic frog family Odontobatrachidae

**DOI:** 10.1186/s12862-015-0346-9

**Published:** 2015-04-19

**Authors:** Michael F Barej, Johannes Penner, Andreas Schmitz, Mark-Oliver Rödel

**Affiliations:** Museum für Naturkunde Berlin, Leibniz Institute for Evolution and Biodiversity Science, Invalidenstrasse 43, D-10115 Berlin, Germany; Department of Herpetology and Ichthyology, Natural History Museum of Geneva, CP 6434, 1211, Geneva 6, Switzerland

## Abstract

**Background:**

Correct species identification is crucial in different fields of biology, and in conservation. The endemic West African frog family Odontobatrachidae currently contains a single described species, *Odontobatrachus natator*. From western Guinea to western Côte d'Ivoire it inhabits forests around waterfalls or cascades. Based on more than 130 specimens from 78 localities, covering the entire distribution, we investigated the molecular diversity of these frogs.

**Results:**

Our analyses included mitochondrial and nuclear genes, with a concatenated alignment of 3527 base pairs. We detected high level of genetic differentiation with five distinct lineages or operational taxonomic units (OTUs). These OTUs were also identified by two different species delimitation approaches, Generalized Mixed Yule Coalescent (GMYC) and cluster algorithm. All OTUs occur in parapatry in the Upper Guinean forests. One OTU, assigned to the “true” *Odontobatrachus natator*, covers the largest distribution, ranging from the border region of western Sierra Leone-Guinea to eastern Liberia. Two OTUs are restricted to western Guinea (Fouta Djallon and foothills), while two others occur in eastern Guinea and the border region of Guinea-Liberia-Côte d'Ivoire. The OTU representing *O. natator* consists of two divergent subclades: one restricted to the Freetown Peninsula (Sierra Leone) and the other covering all populations further inland. Environmental niche models indicated that the restricted Freetown Peninsula population is separated by unsuitable habitat from remaining populations.

**Conclusion:**

Geographic isolation of OTUs and molecular differences comparable to species level differentiation in other frog families indicate that *O. natator* contains cryptic species diversity. Respective distribution patterns most probably resulted from repeated changes of forest cover (contraction and expansion) over evolutionary timescales. The survival within forest refugia that have persisted through multiple drier periods and subsequent dispersal during wetter times may best explain the observed geographic distributions of OTUs. According to the IUCN Red List range criteria each OTU should be classified as “Endangered.” If the Freetown Peninsula “*natator”* population is recognized as a distinct species it would warrant recognition as “Critically Endangered.” The identification of cryptic lineages highlights the urgent need to protect these frogs, all of which are endemic to small areas within the Upper Guinean biodiversity hotspot.

**Electronic supplementary material:**

The online version of this article (doi:10.1186/s12862-015-0346-9) contains supplementary material, which is available to authorized users.

## Background

During field work, biologists usually use morphological characters for species identification as a first approach, especially as vision is our prevailing mode of sensory perception. Investigating animals like birds, insects or anurans, experts often additionally rely on acoustics [1-3], but such expertise is sometimes lacking and species identification can remain uncertain. However, correct species identification is of high importance for both basic and applied research in the field including agricultural science of pest species [4], medical treatment [5], ecological studies [6], and conservation efforts [7-9]. A particular problem for correct species delimitation and identification are so-called cryptic taxa [10], herein referring to superficially (morphologically) indistinguishable lineages. Understudied cryptic species complexes may lead to inaccurate scientific results in studies of community ecology [11,12], population assessments in economically exploited species [13], or conservation decisions aimed at retaining phylogenetic diversity [14].

Improved methodologies, such as molecular and acoustic techniques, make biologists increasingly aware of cryptic species and related problems (e.g. the lack of reliable morphological features for identification [10,15,16]). Cryptic taxa are not restricted to taxonomic groups or biogeographic regions [17]. These may comprise inconspicuous taxa in pathogenic fungi [18], bryophytes [19], insects [20], small mammals [15,21], birds [1], or reptiles [22], but may also include charismatic organisms such as hammerhead sharks [23], lemurs [24], giraffes [25], and elephants [26].

Quite often complexes of cryptic species include lineages with wide distributions. Molecular analyses provide a reliable and quick approach to search for geographically circumscribed lineages and may facilitate discerning minor but diagnostic morphological differences between these lineages [8,19,23,27,28].

Anuran amphibians have been recognised as a group with various examples of cryptic species [29,30]. Many frog species are superficially similar in morphology and possible minor differences can be difficult to observe in the field [31,32].

*Odontobatrachus natator* (Boulenger, 1905) is the only species of the frog family Odontobatrachidae, a recently discovered lineage endemic to Upper Guinea, West Africa [33]. These frogs depend on streams with strong currents and cascades or rapids in forested areas. They occur at mid elevations and have a wide but patchy distribution, ranging from western Guinea to western Côte d'Ivoire [34-39]. Upper Guinean montane forests are already known to contain cryptic species in various taxonomic groups including bats [40-42] or rodents [43]. Populations of *O. natator* are known to vary in colouration and shape of glandular dorsal ridges [37,44], and Barej et al. [33,45] recovered unexpectedly high genetic variance between populations. Following these preliminary molecular findings, we conducted a genetic analysis based on more than 130 samples of *Odontobatrachus natator* covering its entire known range to access molecular variation within this family. We also modelled the potential geographic limits of recognised genetic lineages to delimit their potential distributions. Additionally, we calculated the IUCN Red List criteria “Extent of Occurrence” and “Area of Occupancy” to categorize the potential threat status for each lineage as a basis for conservation decisions.

## Results

### Phylogenetic relationships and diversity

**Figure 1:**
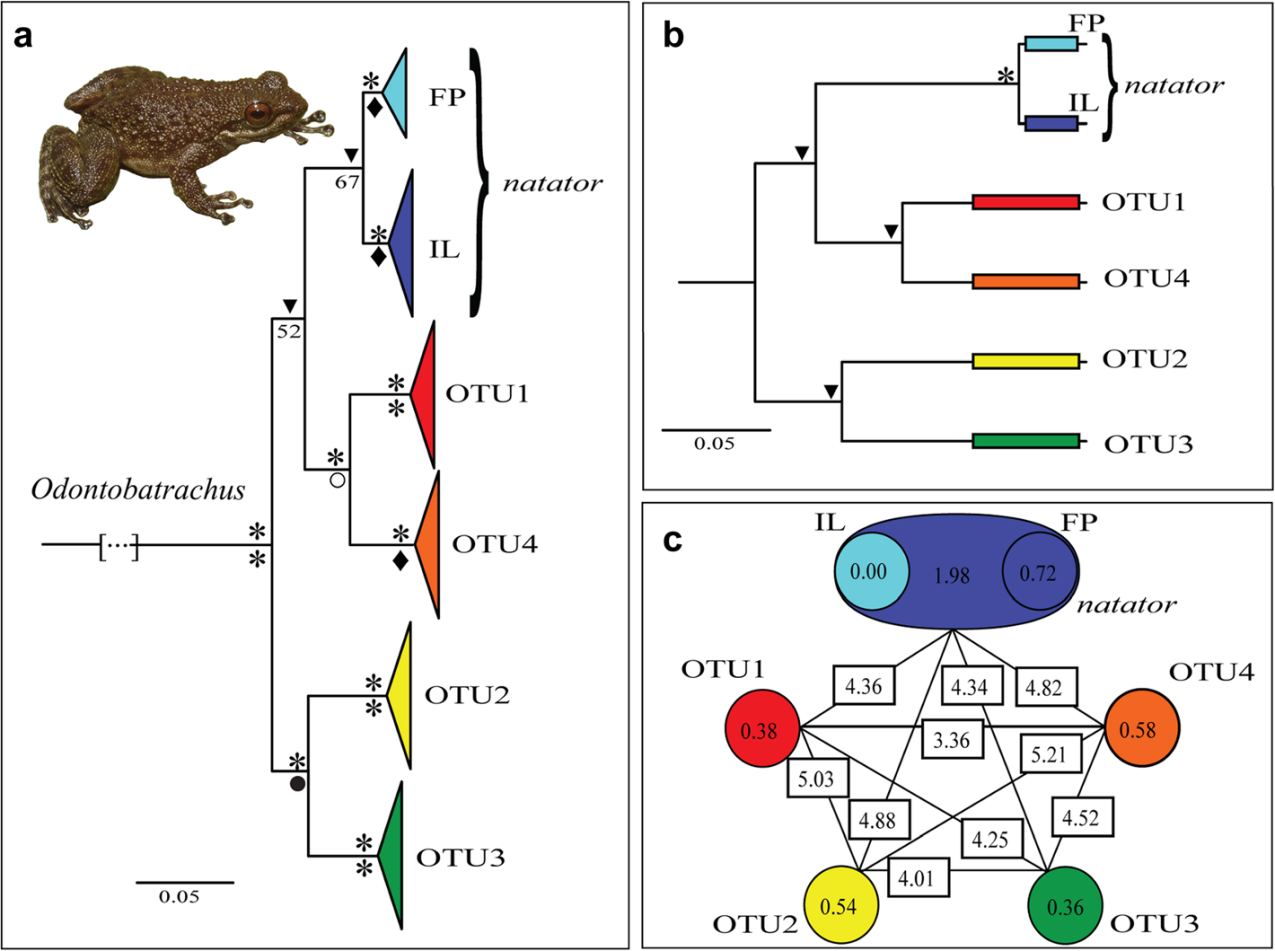
Trees and uncorrected p-distances of *Odontobatrachus*.** (a)** Tree resulting from partitioned Bayes and ML analyses of mitochondrial genes *16S*, *12S*, *cytb* and nuclear genes *RAG1*, *SIA* and *BDNF* (outgroups not shown). **(b)** Species tree from mitochondrial DNA data from the Bayesian Inference of Species Trees (STARBEAST). Support values for (a) and (b) are provided as Bayesian posterior probabilities (above branch; PP: * = 1.00; 0.95 ≥ ▼ ≥ 0.99) and Boostrap support values (below branch; BS: * = 100%; 90 ≥ ♦ ≥ 99; 80 ≥ ● ≥ 89; 70 ≥ ○ ≥ 79). **(c)** Mean uncorrected *16S* p-distances between the five OTUs (rectangles) and maximum p-distances within each OTU (circles). Subclades in *natator* refer to the Freetown Peninsula, Sierra Leone (FP, light blue) and remaining *natator* population further inland (IL, dark blue). Remark: within p-distances in *natator*-subclades possess distinctly lower values than the whole OTU *natator*. Minimum, maximum, mean values and standard deviation of p-distances between and within OTUs are provided in tables 1 and 2.

**Figure 2:**
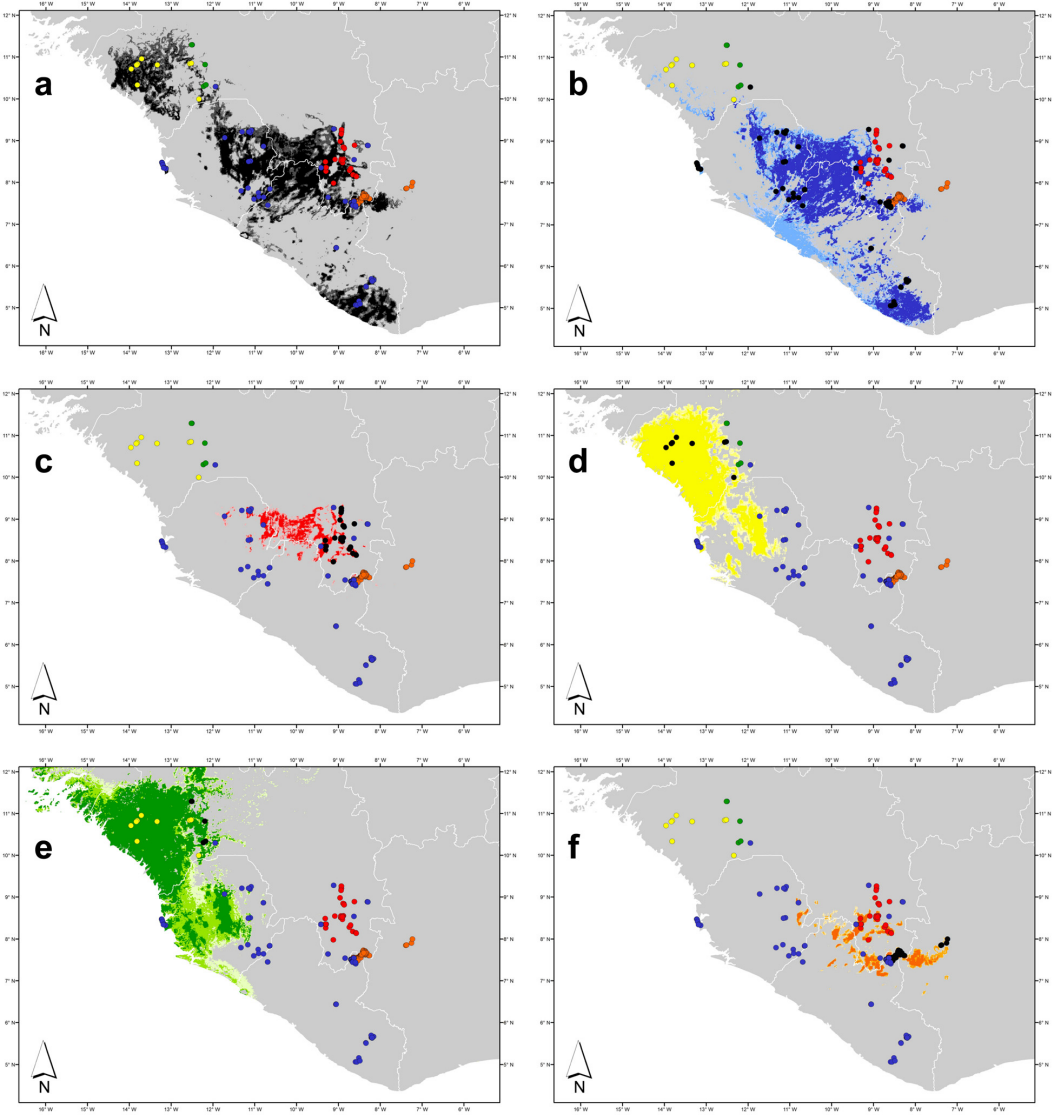
Environmental niche modelling (ENM) maps of genetically confirmed records in the genus *Odontobatrachus*. Increasing intensity in colouration represents the geographical extent of three different models, predicting the potential distribution (light colour = maximum; normal colour = mean; dark colour = minimum; niche parameters see Table 5). ENMs refer to potential distribution of: **a**) Odontobatrachidae (OTUcomb: *natator* + OTU1-4; light grey to black); **b**) OTU *natator* (light blue to intensive blue); **c**) OTU1 (light red to intensive red); **d**) OTU2 (light yellow to intensive yellow); **e**) OTU3 (light green to intensive green); **f**) OTU4 (light orange to intensive orange); sampled localities of each OTU are marked in black.

Results of two different phylogenetic methods, Maximum Likelihood (ML) and Bayesian Inference (BI), both were congruent in recovering the same five major OTUs. The tree topology resulting from 3527 bp concatenated mitochondrial and nuclear genes, with respective node support values, is shown in Figure 1a (the expanded tree and a map showing distribution of OTUs in Upper Guinea is provided in Additional file 1). Relationships among these OTUs were not all strongly supported in the ML analysis. The five OTUs formed two divergent major clades, one consisting of three clades from the central and eastern parts of the distribution range, while the second major clade is distributed exclusively in western localities (Figure 2a). A congruent topology has been uncovered in the species tree approach (Figure 1b).

The number of candidate species identified by SpeciesIdentifier (approach 1, see below) depended on the applied threshold values. The number of recognised clusters increased with decreasing threshold values (Additional file 2). Threshold values differed between analysed mitochondrial genes suggesting different evolutionary rates, with *12S* being the slowest and *cytb* the fastest one. A total of five clusters, corresponding to clades (herein OTUs) in the phylogenetic analyses, as well as the subdivision within *natator*, are recognized.

Mean uncorrected p-distances of the *16S* rRNA between the five OTUs, ranged between 3.36-5.21% (Figure 1c). Summary of *16S* rRNA uncorrected p-distances within and between OTUs are provided in Tables 1 and 2. Remarkably, *natator* possessed a sub-division of haplotypes dividing the coastal population from the Freetown Peninsula in Sierra Leone (FP) and the remaining population further inland (IL). This pattern was reflected in intraspecific p-distances within OTUs ranging from 0.36-0.58% for OTU1-4 and 1.98% in *natator* (Figure 1c). Intraclade values within each subclade of *natator* were 0% for FP and 0.72% for the more widespread IL, respectively. A subdivision of *natator* in two subclades FP and IL corresponded to the increase of recognized clusters in SpeciesIdentifier. The GMYC model (approach 2, see below) identified a robust number of possible OTUs both in the outgroup and in the ingroup (Odontobatrachidae, *Odontobatrachus*). Depending on the priors used to construct the ultrametric tree in BEAST we found a range from 9–14 OTUs in the complete dataset including the four included outgroups (Additional file 3). There was no significant difference between the single- and multiple-threshold approaches; both methods identified either 6 or 7 clusters and between 2–7 singletons. The Yule model with the lognormal relaxed clock produced the lowest number of total groups both with the single- (9) and multiple-threshold (12) approaches, but this approach also produced the largest confidence limit interval in the single-threshold approach (3–13), while all other single approaches produced the same very small confidence interval for the total recovered groups (10–11). Concentrating on the taxa within *Odontobatrachus* we found that exactly the same OTUs postulated by the other applied methods were identified in the single-threshold approaches, while the multiple-threshold approaches predicted further splits within those previously identified OTUs leading to few singletons (Additional file 4). Dating results based on substitution rates point to very young speciation in Plio-Pleistocene times for the genus *Odontobatrachus* (Additional file 5). However, exact timing of splits should be regarded with caution until more recent calibration points are available, enabling a more accurate dating approximation. The original information for the type locality of *Odontobatrachus natator* (Boulenger, 1905) stated just “Sierra Leone”. However, as only one OTU occurred in all known samples from Sierra Leone the assignment of the name *natator* (colour code: blue) to this particular OTU was without doubt. Although ENMs indicated potential distribution of additional OTUs in Sierra Leone, their presence is unlikely (see below). Sampled localities of OTU *natator* showed the widest distribution in the genus ranging from western Guinea through Sierra Leone to eastern Guinea and eastern Liberia (Figure 2b). OTU1 (colour code: red) referred to sites in the Simandou Range and the Massif du Ziama in south-eastern Guinea (Figure 2c). OTU2 (colour code: yellow) and OTU3 (colour code: green) covered haplotypes entirely located in western Guinea (Figures 2d, e). OTU4 (colour code: orange) was based on haplotypes from the Nimba Mountains and a few adjacent elevations in south-eastern Guinea and northeastern Liberia, as well as populations from Mont Sangbé National Park in western Côte d'Ivoire (Figure 2f). Phylogenetically, the OTUs from west (OTU2, OTU3) and east (OTU1, OTU4) were closely related, while *natator* was placed closer to eastern OTUs (Figure 1a). However, the *natator* placement was not strongly supported in this or other concatenated analyses.

**Table 1:** **Uncorrected p-distances between Odontobatrachidae OTUs based on 567 bp of the** ***16S*** **rRNA gene**

	***natator***	**OTU1**	**OTU2**	**OTU3**	**OTU4**
*natator*	---	4.36 ± 0.21 (1216)	4.88 ± 0.19 (1216)	4.34 ± 0.20 (418)	4.82 ± 0.27 (1596)
OTU1	3.74 - 4.87	---	5.03 ± 0.14 (320)	4.25 ± 0.13 (352)	3.36 ± 0.22 (1344)
OTU2	4.50 - 5.40	4.86 - 5.41	---	4.01 ± 0.11 (110)	5.21 ± 0.17 (420)
OTU3	3.97 - 4.88	3.99 - 4.53	3.79 - 4.15	---	4.52 ± 0.16 (462)
OTU4	3.40 - 5.40	2.89 - 3.97	4.60 - 5.55	4.17 - 4.98	---

Given are minimum to maximum p-distance values (lower left triangle) for all five major OTUs, *natator* and OTU1-4 and mean values with standard deviation and in brackets the number of comparisons (upper right triangle).

**Table 2:** **Uncorrected intra-OTU p-distances in Odontobatrachidae OTUs based on 567 bp of the** ***16S*** **rRNA gene**

	**min**	**max**	**mean**	**SD**	**N**
OTU1	0.00	0.38	0.18	0.15	496
OTU2	0.00	0.54	0.20	0.19	45
OTU3	0.00	0.36	0.15	0.15	55
OTU4	0.00	0.58	0.05	0.11	861
*natator* (FP + IL)	0.00	1.98	0.42	0.51	703
*natator* (IL)	0.00	0.72	0.27	0.21	630
*natator* (FP)	0.00	0.00	0.00	---	1

Given are minimum (min), maximum (max), standard deviation (SD) and sample size (N = number of pairwise comparisons) for all five major OTUs, *natator* and OTU1-4, as well as the subdivision within *O. natator* following molecular and distributional subdivision (FP, IL).

### Haplotype networks

**Figure 3:**
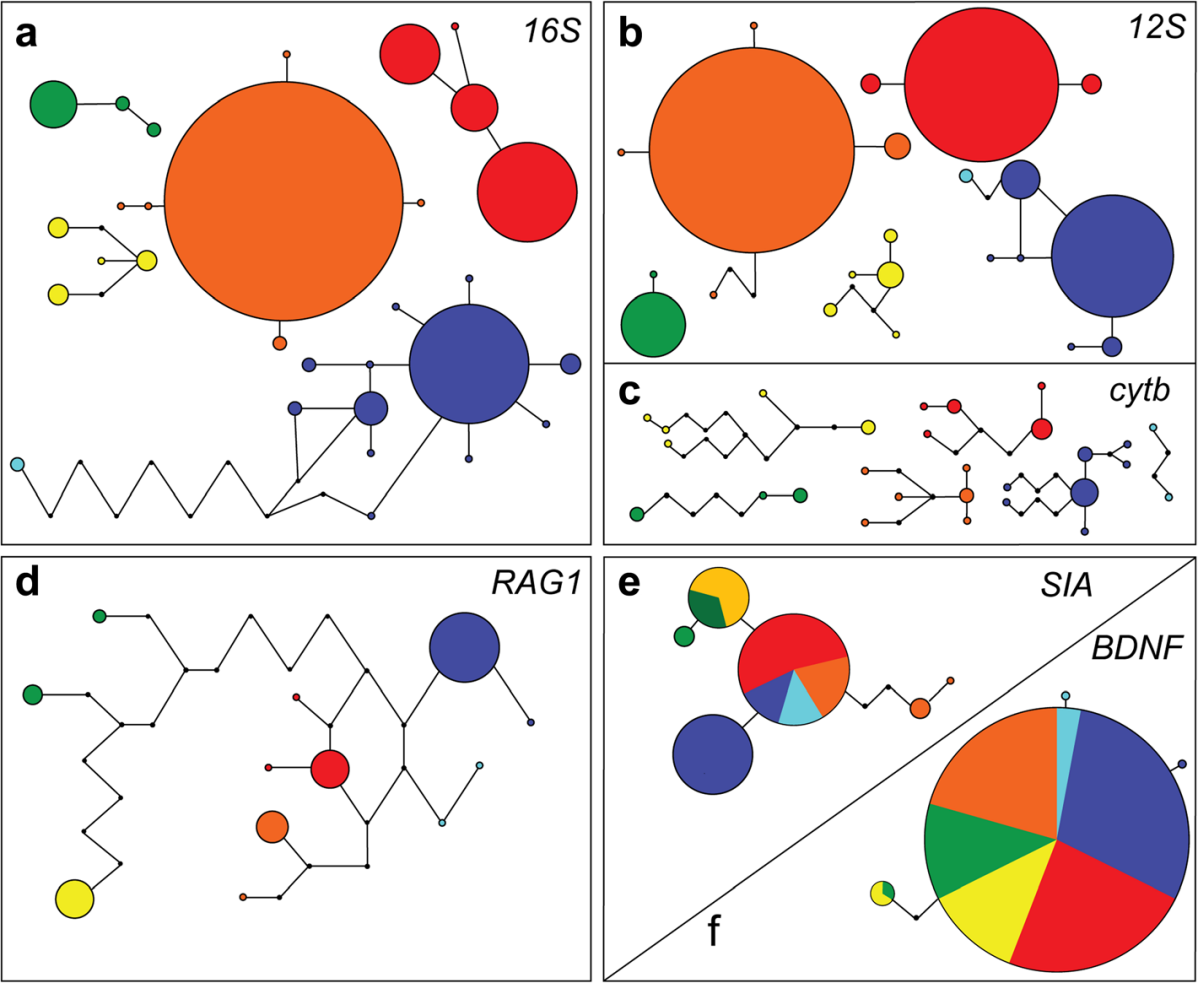
Parsimony networks of haplotypes in Odontobatrachidae. Networks corresponding to mitochondrial genes *16S* (**a**; N = 133), *12S* (**b**; N = 123), *cytb* (**c**; N = 39) and nuclear genes *RAG1* (**d**; N = 38) and *SIA* (**e**; N = 47) and *BDNF* (**f**; N = 39) sequence variation from analysed samples. The colours correspond to distribution maps (Figure 2) and trees (Figure 1): *natator* (IL; dark blue), *natator* (FP; pale blue), OTU1 (red), OTU2 (yellow), OTU3 (green) and OTU4 (orange). Mitochondrial genes **(a-c)** show un-associated sub-networks and two nuclear genes **(e, f)** show almost identical haplotypes. The size of the circles is proportional to the number of alleles for each gene.

Differences in haploid genotypes of OTUs and their relationships were assessed with unrooted haplotype networks, showing one-step mutations; identical sequences were pooled into a single terminal. Numbers of analysed sequences and uncovered haplotypes were as follows (N_samples_/N_haplotypes_): mitochondrial genes *16S* (133/30), *12S* (123/22), *cytb* (39/28), nuclear genes *RAG1* (38/12), *SIA* (47/6) and *BDNF* (39/4). Haplotype networks of mitochondrial genes (*12S*, *16S*, *cytb*) showed a clear separation of clades and partitioning of haplotypes to a degree of forming distinct and un-associated sub-networks for all five OTUs (Figure 3a-c). Comparable to the results above, *natator* formed two distinct subclades. These were un-associated in *cytb* (Figures 3c), divided by a minimum of nine mutation steps in the *16S* gene (Figure 3a) and even in the slowest mitochondrial gene, *12S*, FP samples were separated from remaining *natator*-haplotypes (Figure 3b). Concerning nuclear markers, solely the *RAG1* gene exhibited a differentiation and privatization of the five OTUs, even separating FP haplotypes in *natator* from remaining localities (Figure 3d). In contrast, *BDNF* and *SIA* showed little variation and a large overlap of shared haplotypes of the five OTUs and within *natator* (Figure 3e, f).

### Diagnostic nuclear sites

Four sites in the *RAG1* gene and a single site in *SIA* supported two major groupings and distinguished the western OTU2 and OTU3 from more eastern *natator*, OTU1 and OTU4. Additionally, the five OTUs were defined in nuclear genes by a few deviating sites only.

### Environmental niche modelling

Overall the ENMs performed well, with training AUC values of 0.9915-0.9985 and test AUC values of 0.9906-0.9982 in individual OTUs and OTUcomb (Additional file 6). Only in OTUcomb and *natator* all parameters contributed to ENMs (Additional file 7). Highest contributions in OTUs were as following: in OTUcomb highest precipitation value or wettest month (prec30_max 43.96%) and total annual precipitation (prec30_sum 28.95%) had highest predictive power; in OTU *natator* highest precipitation value (wettest month; prec30_max 34.83%) and total annual precipitation (prec30_sum 34.64%); in OTU1 total annual precipitation (prec30_sum 30.07%) and lowest precipitation value (driest month; prec30_min 22.34%); in OTU2 standard deviation of the precipitation (prec30_std 49.90%) and percentage of bare ground (MODIS; bare_4x4 25.75%); in OTU3 standard deviation of precipitation (prec30_std 46.14%) and percentage of bare ground (MODIS; bare_4x4 38.12%); in OTU4 lowest precipitation value (driest month; prec30_min 31.00%), elevational variance calculated from the SRTM30 data set using a 9x9 moving window (srtm_v_ln_9x9 25.63%) and highest precipitation value (wettest month; prec30_max 24.39%). Consequently, parameters with highest contribution in OTU2 and OTU3 were identical (OTU2/OTU3: percentage of bare ground and standard deviation of precipitation), while OTUs *natator*, OTU1 and OTU4 had one parameter in common respectively (OTU1/OTU4: driest month; OTU1/*natator*: total annual precipitation; OTU4/*natator*: wettest month).

ENMs of potential distribution of the family Odontobatrachidae (OTUcomb) revealed an area smaller than just superposed ENMs of all separate OTUs (Figure 2a). However, it showed that samples included herein covered the entire distribution of this family. Considering ENMs the border area between Sierra Leone-Guinea-Liberia might inhabit up to three OTUs of odontobatrachid frogs, however records from that area lack so far. ENMs of *natator* predominantly covered the border area of these three countries (westwards through Sierra Leone into western Guinea), and south-eastern Liberia with some extensions into western Côte d'Ivoire (Figure 2b). Moreover, a distinct gap of apparently unsuitable area separated *natator* localities around Freetown, Sierra Leone from other populations further inland Figure 2b). ENMs of OTU1 covered parts of south-eastern Guinea and westwards into Sierra Leone (Figure 2c). ENMs of OTU2 and OTU3 showed a largely overlapping potential area of distribution in western Guinea and Sierra Leone (Figure 2d, e). ENMs of OTU4 covered areas in south-eastern Guinea/northern Liberia and further westwards reaching eastern Sierra Leone (Figure 2f). Generally, ENMs of OTU1 and OTU4 showed very little or no overlap in their potential distribution with OTU2 and OTU3. Concerning sampled localities, only *natator* possessed areas overlapping distribution ranges of eastern OTUs (OTU1 and OTU4) in south-eastern Guinea and reaching easternmost distribution boundaries of western OTUs (OTU2 and OTU3) in western Guinea, respectively (Figure 2).

### Conservation status

Both applied species delimitation methods revealed 5–6 OTUs within *Odontobatrachus*. We examined their potential conservation status, at first in a more conservative approach (5 OTUs). In a second step we assess the potential conservation status of two lineages within “*natator”*. Following the IUCN Red List geographic range criterion the calculated EOOs classified OTU1 and OTU2 as “Vulnerable.” OTU3 and OTU4 as “Endangered” and only *natator* as of “Least Concern” (Table 3). In contrast, AOO calculations depicted ranges classifying all five OTUs as “Endangered.” If both *natator* subclades are recognised as distinct species, the Red List category for the Freetown Peninsula subclade would change to “Critically Endangered” due to its low AOO (Table 3).

**Table 3:** **Summary of IUCN Red List criteria for Odontobatrachidae OTUs**

	**AOO**		**AOO**	
	**km** ^**2**^	**RL category**	**km** ^**2**^	**RL category**
OTU1	7.797	Vulnerable	104	**Endangered**
OTU2	12.673	Vulnerable	40	**Endangered**
OTU3	1.318	**Endangered**	20	**Endangered**
OTU4	2.529	**Endangered**	156	**Endangered**
*natator*	180.231	Least Concern	224	**Endangered**
*natator* (IL)	132.175	Least Concern	204	**Endangered**
*natator* (FP)	34	**Critically Endangered**	20	Endangered

IUCN Red List (RL) range criteria for all OTUs and the two *natator* subclades, resulting from calculation of the Extent of Occurrence (EOO) and Area of Occupancy (AOO) [126]. Decisive category marked in bold letters. Additionally, results are provided for both *natator* subclades (Freetown Peninsula = FP; inland = IL) independently in case they represent distinct species.

## Discussion

Taxonomic decisions in cryptic species complexes are usually based on subtle morphological characters. Morphological congruence not only impedes species recognition, due to absence of striking differences, but the whole description process itself as statistically exploitable numbers of vouchers are needed to recognize differences in otherwise overlapping morphometrics. Because genetic divergence accompanied by morphological similarity is not uncommon, cryptic speciation either remains overlooked [28,46] or species diagnoses focus exclusively on molecular markers [47].

Our analyses of the West African torrent-frog *Odontobatrachus natator,* presumed to be widely distributed, uncovered a cryptic diversity within this unique evolutionary lineage [33]. Based on analyses of concatenated mitochondrial and nuclear loci, we recognize five OTUs in the family Odontobatrachidae. Both applied species delimitation approaches indicate the presence of five or six candidate species within *Odontobatrachus*, corresponding to OTUs in the phylogenetic analyses, respectively. The mitochondrial *16S* gene is commonly used for barcoding approaches in anuran amphibians [29,30,48], and uncorrected p-distances recognized between OTUs are comparable to species level differences in non-related frog genera and families thus pointing to cryptic speciation in *Odontobatrachus* (min-max: 2.89-5.55%; Table 1; compare [2,30,49-54]). Recognized p-distances within each OTU were considerably lower than 0.72% (Table 2).

Mitochondrial genes demonstrated utility for determining lineages of geographically separated OTUs [49,55] and all five OTUs have discrete *16S*, *12S* and *cytb* haplotypes. Of the nuclear genes, solely *RAG1* distinguished all five OTUs by discrete haplotypes. Geographically adjacent OTUs have been pooled in two of three nuclear genes (*RAG1*: 4 bp; *SIA*: 1 bp) supporting a geographical split of the western (OTU2, OTU3) against the remaining OTUs in the Upper Guinean forests (Figure 3). Differences in the level of separation in nuclear genes may be due to incomplete lineage sorting at each single nuclear loci or different evolutionary rates [56,57].

The unexpected recognition of five distinct OTUs raised the necessity to assign the officially described taxon *Odontobatrachus natator* (Boulenger, 1905) to one of them. Despite the fact that ENMs indicated the potential occurrence of all OTUs in Sierra Leone, all sampled localities from Sierra Leone belonged genetically to a single OTU. The type locality of *O. natator* is Sierra Leone, consequently the respective OTU was assigned to the nominate taxon (Figure 2). Generally, OTUs showed a parapatric distribution in Upper Guinea and distribution patterns in the family Odontobatrachidae are now regarded as follows (Figure 2a): *O. natator* has the widest range and is present in lowlands to mid-altitudes from the southern edge of the Fouta Djallon in western Guinea to eastern Liberia and southeastern Guinea (200–1350 m a.s.l.); OTU1 occurs in lowlands to mid-elevations north of the Nimba Mountains in south-eastern Guinea (e.g. Simandou Range, Massif du Ziama, Mont Bero; app. 450–1300 m a.s.l.); OTU2 occurs in lowlands in western Guinea (the Fouta Djallon massif and its western extensions into the Télimélé region; app. 100–650 m a.s.l.); OTU3 likewise occurs in western Guinea but at higher elevations (Fouta Djallon; app. 650–900 m a.s.l.); OTU4 occurs in lowlands to mid elevations in the border area Guinea-Liberia-Côte d'Ivoire and further east (Mount Sangbé in western Côte d'Ivoire, the Nimba Mountains and adjacent Mount Gangra; app. 400–1300 m a.s.l.). Consequently, geographic separation and genetic distinctness of OTUs at the species-level, identify *Odontobatrachus natator* as a complex of cryptic species.

Potential geographic ranges based on ENMs exceeded the known distribution ranges in four of five OTUs; the only exception is *natator* (Figure 2). OTU1 and OTU4 could be widely distributed in eastern Guinea and the border region of Sierra Leone-Guinea-Liberia, while OTU2 and OTU3 could occur in central Sierra Leone and even enter Guinea-Bissau in the case of OTU3 (Figure 2). However, we believe that these predictions are unlikely. Given our own dense sampling of the entire Upper Guinea forest zone, it is reasonable to assume that the observed ranges are close to the known ranges of all five OTUs. Field observations coarsely explain the occurrence of *Odontobatrachus* by two factors: 1) forest cover and 2) slopes (in different elevations) with fast-flowing streams of various sizes, including waterfalls and cascades. As various areas in the Upper Guinean forests are apparently suitable to several OTUs and geographic barriers, such as large rivers or mountain chains do not exist in this area ([58] the one exception, the Kolenté river, bordering Guinea and Sierra Leone, might separate *natator* and OTU2), competitive exclusion due to niche occupation by *natator* may shape the distributions of OTU1-4 [59].

OTUs more closely related to each other are likewise spatially closer: a western clade is present in the Fouta Djallon highlands and its western extensions (OTU2, OTU3), and the central-eastern clade occurs in the Guinea Highlands, running from southern Fouta Djallon through northern Sierra Leone and Liberia to western Côte d'Ivoire (*natator*, OTU1, OTU4; Figure 2a). A potential scenario responsible for such a distribution might be the fluctuations in African palaeo-environments and repeated expansion and contraction of suitable forested habitat for species such as those of the family Odontobatrachidae [60-62]. The refuge theory aims at explaining how such forest refugia, especially montane forests, have boosted speciation in forest dependent species during past arid epochs [63-65]. The role of Upper Guinean forests as refugia is uncontroversial [65,66]. But, knowledge concerning the exact historical process (timing and geography) of West African refugia remains scarce [67]. In particular the existence and geographic position of so-called micro-refugia, small forest areas outside postulated larger refugia, is unclear. However, these may be particularly important for the persistence of a large portion of regional biodiversity [68].

Close relationships between *Odontobatrachus* OTUs cause a lack of resolution in slow-evolving nuclear genes (Figure 3e, f). Despite a clear resolution, likewise the comparatively low number of changes in fast evolving mitochondrial genes *12S* and *16S* points to rather recent splits, and the following speciation scenario seems most likely. While calculations of node ages should be regarded with caution until more recent calibration points are available, calculated node ages younger than 3 Ma support very young (Plio-Pleistocene-)speciation events in the genus *Odontobatrachus* (Additional file 5). Torrent frogs may have evolved in the Central Guinea Highlands (max. app. 1950 m a.s.l.), and then colonised westwards to the Fouta Djallon (max. app. 1500 m a.s.l.) and eastwards to the Simandou Range-Nimba Mountains (app. 1700 m a.s.l.), always settling along fast running streams in forests (Additional file 8). For the Plio-Pleistocene several aridity-dominated periods are known [61] and while open habitats dominated the landscape in Upper Guinea, torrent frogs have probably only prevailed in isolated montane forests as it is also assumed for other forest-dependant groups [67]. The Fouta Djallon as well as the Simandou Range-Nimba Mountains are known to comprise many endemic species [69-72] and both are assumed to play a role as Upper Guinean forest refugia [66,71,73]. A subsequent turn of forest expansion and contraction might have resulted in sister relationship of OTUs in eastern and western Upper Guinea (east: OTU1/OTU4 and west: OTU2/OTU3; Figures 1a and 2a). The Loma Mountains-Tingi Hills area (app. 1900 m a.s.l.) in Sierra Leone might have served as the refuge for *natator* in central Guinea Highlands (Additional file 8). The latest expansions of forests and increase of suitable habitats was apparently exploited best by *natator* as this OTU shows the widest distribution. Probably a population of *natator* was likewise pushed back to coastal elevations of the Peninsula Mountains (app. 900 m a.s.l.) during relatively cold climates, resulting in molecular divergence. Today, both *natator* subclades are separated by unsuitable habitat due to lack of forest cover that might result from environmental changes in more recent times [74,75] and not least from anthropogenic deforestation, e.g. during colonization events [76] and before.

Differing dispersal patterns of *Odontobatrachus* OTUs, showing differences in size of distribution areas, could result from minor differences in: habitat requirements, ecological adaptations or persistence in more widespread micro-refugia. Such ecological data should be searched for in future studies of this genus. However, ENMs revealed that closer related OTUs (*natator*/OTU1/OTU4 *vs*. OTU2/OTU3) share important factors shaping their potential distribution which play a minor role for the sister clade (Additional file 7). Generally, precipitation played an important role in shaping distribution of all OTUs although important precipitation parameters differed between OTUs (OTU1/OTU4: driest month; OTU1/*natator*: total annual precipitation; OTU4/*natator*: wettest month; OTU2/OTU3: standard deviation of precipitation; Additional file 7).

In conclusion, the assumed monospecific West African frog family Odontobatrachidae obviously contains several undescribed species. Distribution patterns provide first insights into the subdivisions of the Upper Guinean forest refugium, but knowledge on the ecological requirements leading to the present distribution patterns of these frogs is incomplete and requires further investigation to support our hypotheses.

### Conservation concern

Amphibian declines occur around the world and in all major habitat types, with forest species showing highest losses [27]. West Africa represents a distinct biodiversity hotspot [58,77]. Its major threats are rapid deforestation, often including degradation and fragmentation, and the increase of agricultural encroachment and mining in Upper Guinean Mountains [78-85]. Taxa with narrow habitat niches are highly threatened by habitat loss and fragmentation effects [86], and because of their dependence on fast flowing streams in forested areas this applies to the West African torrent frogs. At present, *Odontobatrachus natator* has been assigned the IUCN Red List category “Near Threatened“ [87], with a trend of decreasing populations. However, our results indicate that the different OTUs represent distinct species, and thus should then be classified as “Endangered.” If the two subclades of *natator* are recognized as distinct species, the Freetown Peninsula population would require the Red List category “Critically Endangered”. Today’s conservation efforts should concern distinct genetic lineages within species [88-90] and consequently should be applied to *O. natator* s.l. as well.

Although Guinea exhibits the highest diversity of the family Odontobatrachidae, with all five OTUs, the network of protected areas is poor [91]. Considering the distribution of this family, only three OTUs occur in protected areas (herein maintained as National Parks and Biosphere Reserves; Additional file 9). However, even in protected areas threats to herein defined OTUs are evident: *natator* (Sierra Leone: Gola Forest National Park, Western Area Peninsula Forest Reserve - urban growth; Liberia: Sapo National Park - stone mining), OTU1 (Guinea: Massif du Ziama Biosphere Reserve - agricultural encroachment, timber exploitation), OTU4 (Guinea/Liberia/Côte d'Ivoire: Mount Nimba Strict Nature Reserve - mining [92]; Côte d'Ivoire: Mont Sangbé National Park - current status unclear after recent political crisis in Côte d'Ivoire). While both *natator*-subclades occur in protected areas, OTU2 and OTU3 (Guinea: Fouta Djallon - logging of gallery forests, local population and tourist activities [39]) are entirely unprotected at present.

Almost everywhere throughout its distribution range *Odontobatrachus natator* s.l. occurs sympatrically with *Conraua alleni*, the latter inhabiting more slowly-running parts of streams. While *C. alleni* is already listed as “Vulnerable” [87] this classification is probably insufficient as high genetic diversity indicates another cryptic complex ([87]; Barej et al. unpubl. data). Consequently, any conservation effort concerning *Odontobatrachus* could also maintain lineages of the *C. alleni* complex*,* as well as other forest and stream dependent taxa of other taxonomic groups, not yet in focus of any more in detail research activities.

## Conclusions

A large-scale molecular assessment of the West African endemic family Odontobatrachidae throughout its distribution range revealed the presence of distinct Operational Taxonomic Units (OTUs). Bayesian methods and ML uncovered five OTUs, which show a parapatric distribution and slight differences in parameters contributing to respective environmental niche models. OTUs most likely represent distinct species and challenge the monospecific status of Odontobatrachidae. The assigned nominate form *Odontobatrachus natator* revealed two subclades in both analyses, which are geographically separated by unsuitable habitat. Following IUCN Red List criteria all five OTUs should be classified as ‘Endangered’ if later recognized as distinct species. Only two OTUs currently occur in protected areas, while all are endangered through habitat loss. Knowledge of forest refugia within the Upper Guinean forests is scarce but the distribution pattern of Odontobatrachidae OTUs suggest likely refugia within the Fouta Djallon, Simandou Range-Nimba Mountains, Loma Mountains-Tingi Hills and the Peninsula Mountains.

## Methods

### DNA extraction, amplification and sequencing

A total of 135 samples from 78 localities covering the entire distribution of the family Odontobatrachidae, ranging from Guinea, Sierra Leone, Liberia and Côte d'Ivoire were analysed (map of most important elevations see Additional file 10). Collected frogs were anesthetized with either chlorobuthanol or benzocaine and fixed in 4% formalin or 75% ethanol. Voucher specimens were finally stored in 75% ethanol. Tissues were taken either from fresh specimens collected in the field or preserved museum specimens, either by toe clips, liver or muscle tissue. DNA extraction, amplification, and sequencing follow the methodology of Barej et al. [45]. For quality assurance we sequenced both directions of the amplified PCR product (using an external vendor, Macrogen). Available samples were barcoded for two standard mtDNA markers (*12S* and *16S*) for a preliminary assignment to major clades, and representatives of preliminary distinct populations were subsequently sequenced for three nuclear (Seven-in-Absentia [*SIA*], Recombination Activation gene 1 [*RAG1*] and Brain-derived neurotrophic factor [*BDNF*]) and one additional mitochondrial coding gene (cytochrome b gene [*cytb*]). Respective primers are given in Barej et al*.* [45]. A full list of samples, their museum collection number and locality data as well as respective GenBank [93] numbers (KP005071-KP005450) are given in Additional file 11.

### Phylogenetic analysis

Sequences were checked for reliability using the original chromatograph data in the program BioEdit [94], aligned using ClustalX [95] and the alignment checked by eye. Protein coding partitions of mitochondrial and nuclear genes (*cytb*, *BDNF, SIA*, *RAG1*) were translated to amino acids with the program TranslatorX [96] to set codon positions and confirm absence of stop codons. The final alignment of all six genes, including nuclear and mitochondrial loci, consisted of 3527 base pairs. Sequence lengths were as following: 383 bp of *12S*, 567 bp of *16S*, 576 bp of *cytb*, 675 bp of *BDNF*, 396 bp of *SIA*, 930 bp of *RAG1*.

Two techniques for phylogenetic estimation were applied: Bayesian Inference (BI; MrBayes, 3.21 x64; [97,98]) and Maximum Likelihood (ML; RAxML 7.0.4; [99] using the rapid hill climbing algorithm following Stamatakis et al*.* [100]). While ML analyses were run under the GTR + G model in RAxML, BI used recognized partition schemes identified with PartitionFinder 1.1.1 [101], models of substitution are provided in Table 4. Additionally we analysed an unpartitioned dataset and a maximally partitioned dataset (Additional file 12). For this purpose, the Bayesian information criterion (BIC) for each gene partition (and respectively codon positions P1, P2, P3) was selected using jModelTest 2.1.4 [102]; models of substitution are provided in Table 4. The next more complicated model (in regard to number of substitutions, among-site variation, number of rate categories for the gamma distribution, considering the mitochondrial or universal code) was implemented when jModelTest results were not applicable. Results of single gene analyses are provided in Additional file 13. Included outgroups (not shown) were *Hyperolius ocellatus*, *Conraua alleni*, *C. goliath* and *Petropedetes juliawurstnerae* (GenBank numbers provided in Additional file 11).

**Table 4:** **Partition schemes and models of substitution**

**8 partitions**			**14 partitions**		
	**substitution model**	**included partitions**		**substitution model**	**included partitions**
1	GTR + G	*12S, 16S; cytb* (P1)	1	TIM2 + G	*12S*
2	F81 + I	*BDNF* (P1), *Rag1* (P1)	2	TIM2 + I + G	*16S*
3	JC	*BDNF* (P2), *SIA* (P2)	3	TIM2 + I + G	*cytb* (P1)
4	K80 + G	*BDNF* (P3)	4	TPM2uf + G	*cytb* (P2)
5	K80 + I	*RAG1* (P2), *SIA* (P1)	5	TIM1 + I + G	*cytb* (P3)
6	K80 + G	*RAG1* (P3), *SIA* (P3)	6	GTR + I	*RAG1* (P1)
7	HKY + G	*cytb* (P2)	7	TIM1 + I	*RAG1* (P2)
8	TrN + I	*cytb* (P3)	8	HKY + I	*RAG1* (P3)
			9	TrNef	*SIA* (P1)
**4 partitions**			10	JC	*SIA* (P2)
1	GTR + G	*12S, 16S, cytb* (P1)	11	TPM2uf + G	*SIA* (P3)
2	JC + 1	*RAG1* (P1), *RAG1* (P2)	12	K80 + G	*BDNF* (P1)
3	HKY + G	*cytb* (P2), *RAG1* (P3)	13	JC	*BDNF* (P2)
4	TrN + I	*cytb (*P3)	14	JC	*BDNF* (P3)

Models of substitution applied in the Bayesian inference (8 partitions; PartitionFinder) and the maximally partitioned dataset (14 partitions; jModelTest). Models of substitution applied in the dating analysis (4 partitions, PartitionFinder).

Support values for the two phylogenetic approaches were calculated. Bootstrap analyses (BS) with 1000 pseudoreplicates evaluated the relative branch support in the ML analysis. Bayesian analyses were run under partitioned schemes for 5 million generations with four chains sampled every 100 generations, with a burn-in of 1000 trees. Clades with posterior probabilities (PP) ≥ 95% were considered strongly supported. Stationarity of Bayes results was checked with Tracer 1.6 [103]. Uncovered strongly supported molecular lineages were defined as operational taxonomic units (OTU) to render distributional delimitation and molecular comparisons possible. Uncorrected p-distances between OTUs and within OTUs were calculated with PAUP* 4.0b10 [104] for the partial *16S* rRNA gene as these values were often used to prove distinctness at the species level [29,30].

### Haplotype networks

Haplotype networks of genealogical relationships for single mitochondrial and nuclear genes were constructed with the software TCS 1.21 [105] with a connection limit of 95% as implemented in the software. Haplotype frequencies were considered and haplotypes coloured according to recognised OTUs. Shared haplotypes are provided as pie charts and colours reflect the proportionate factor of respective integrated main lineages.

### Species tree

A species tree was generated with the software package BEAUti and BEAST 1.7.5 and the implemented approach *BEAST [106,107]. The analysed data set included all samples covering all mitochondrial genes. This approach required *a priori* definition of ‘species’ (referring to herein phylogenetically identified OTUs) which have been entered according to the phylogenetic results. Resulting log files were checked via Tracer 1.5.0 and trees combined with the software LogCombiner 1.7.5 and TreeAnnotator 1.7.5 with 10% of the trees discarded as burn-in. The final trees were visualised with FigTree 1.4.0 [108].

### Species delimitation

The genetic diversity within *Odontobatrachus natator* was examined on the basis of the mitochondrial genes *16S*, *12S* and *cytb* (approach 1 see below) or the concatenated alignment including all mitochondrial and nuclear genes with the exception of the position 2 of the *SIA* codons, since this position turned out to be nearly invariable for the dataset (approach 2). While approach 1 considers overlaps between intra- and interspecific variation, approach 2 seeks to identify groupings on the species-level versus population level resulting from tree shape and branch lengths of a given tree.

In approach 1, OTUs were assessed using the software TaxonDNA 1.7 and the implemented ‘Cluster’ algorithm in SpeciesIdentifier [109]. OTUs, therein termed clusters, are identified according to pairwise distances for sequences within each cluster. We herein reduced the data set to unique haplotypes for each applied gene. Incremental values ranged from 0.5% (exceptionally 0.75 in *12S*), with an increase of 0.5% each step, to a maximum of 4.5%. However, the calculations were stopped if all haplotypes were grouped in a single cluster/OTU. The maximum pairwise distance within recognized OTUs (a putative species-level) should not exceed a given threshold.

In approach 2 we examined the clustering of the ingroup taxa (Odontobatrachidae, *Odontobatrachus*) by detecting the boundaries between species-level and population-level with the GMYC approach [110-112]. GMYC can use either the Yule model (for lineage diversification processes) or the coalescent model (for population-level diversification) and find the respective transition in branching rates between them. We used both the single transition [110] and the multiple transition [112] approaches. Observed branching rates were subjected to a log-likelihood ratio test (LRT) which uses a null-hypothesis suggesting no shift in the branching rate. If shifts are observed in the branching rates, one can assume more than one OTU in the used dataset. For the GMYC analyses we used ultrametric trees that were created with a random starting tree, and the GTR + I + G model of sequence evolution in BEAST [113]. To assure a stable result not influenced by priors we followed the approach of Gehring et al. [114] and created 4 different ultrametric trees which were generated using 1) a Yule model for the tree prior, with a strict molecular clock (fixed to a rate of 1.0), 2) a Yule model with a lognormal relaxed clock, 3) a coalescent model with a strict clock, and 4) a coalescent model with a lognormal relaxed clock. The GMYC web server (The Exelixis Lab: http://species.h-its.org/gmyc/) was used to fit our four trees to both single-transition and multiple-transition GMYC models.

### Dating estimates

Dating was performed with the software package BEAUti and BEAST 1.8.1 [106,107] on a reduced dataset consisting of the complete ingroup (*Odontobatrachus* OTUs) and a single outgroup (*Petropedetes juliawurstnerae*). Additionally, we deleted the slow evolving nuclear genes *SIA* and *BDNF* from the dataset since they had not accumulated enough mutation in the ingroup to show any resolving power (see haplotype networks Figure 3e, f); this lead to a full dataset of 2446 bp of coding and non-coding genes (Additional file 5). Partition schemes were again calculated with PartitionFinder 1.1.1 [101], for identified models of substitution see Table 4.

To accommodate for the comparatively short branch length identified in the full analyses the data were run under a Coalescent speciation model [115] and the lognormal relaxed clock model [116], using a random starting tree. Because of the lack of suitable calibration points for the evolutionary history of the ingroup we modelled informative priors for the mutation rates only, using the published data [117-119] (means of 0.0125 for the combined mitochondrial genes and 0.00262 for the *RAG1* gene, respectively), both with a normal distribution and a relaxed standard deviation of 0.1. To check for inconsistencies between the mitochondrial and the nuclear gene mutation rates, we ran analyses using the mitochondrial priors only as well as priors for both the mitochondrial and nuclear genes together. We ran all analyses for 1.25 billion generations total (5 runs of 250 million generations, with a burn-in of 20 million and 25 million generations for the mitochondrial only and combined mitochondrial-rag1 dataset, respectively). Convergence and mixing of the parameters for each run were checked in Tracer 1.6 [103].

### Environmental niche modelling (ENM)

In order to assess the potential current distribution of recognised OTUs in the Upper Guinea forests, ENMs were calculated. ENM as a statistical modelling tool seeks to determine relationships between species occurrences and environmental parameters within data sets. Based on such correlations, potential distributions can be modelled. We applied ENMs using maximum entropy principles (using the software Maxent 3.3.3.k [120-122]) by comparing values of variables at sites where the species was found against data from randomly chosen sites where the species was absent (background). As incorrect absence information might be counterproductive, a conservative approach based on confirmed presence only was chosen. Maxent is one of the best ENM techniques when using presence-only data [123,124]. A total of 18 continuous parameters (5 environmental, 10 climatic, 2 altitudinal, 1 distance-based; Table 5) were analysed on a 30 arc second grid (app. 1 km^2^) on a continental scale and clipped back to the Upper Guinea forest area within West Africa. Climatic parameters corresponded to average values from 1950 to 2000 (for details see Table 5). Environmental parameters were based on satellite imagery (SPOT4 & MODIS). Altitudinal parameters were converted from a radar derived data set (SRTM). Following Penner et al. [125] a total of 100 ENMs were calculated and replicated using sub-sampling (70% model training and 30% model testing) and finally three average models were derived: maximum, mean and minimum prediction gained. Average 10 percentile thresholds were applied over all ENMs to gain three binomial models from maximum, mean and minimum models. Validation of models was performed with the area under the curve (AUC) criterion, which corresponds to the receiver operating characterising (ROC) curve; a threshold-independent measurement widely accepted for such models [123].

**Table 5:** **Parameters used in the environmental niche modelling (ENM) approach**

	**Category**	**Parameter**	**Description**	**Original source**
1	climate	tmin30_max	highest value of the minimum temperatures	[127]
2	climate	tmin30_min	lowest value of the minimum temperatures	[127]
3	climate	tmin30_std	standard deviation of the minimum temperatures	[127]
4	climate	tmax30_max	highest value of the maximum temperatures	[127]
5	climate	tmax30_min	lowest value of the maximum temperatures	[127]
6	climate	tmax30_std	standard deviation the maximum temperatures	[127]
7	climate	prec30_max	highest precipitation value (wettest month)	[127]
8	climate	prec30_min	lowest precipitation value (driest month)	[127]
9	climate	prec30_std	standard deviation of the precipitation	[127]
10	climate	prec30_sum	total annual precipitation	[127]
11	environment	glc_raw2	vegetation derived from the near-infrared (0.78-0.89 μm) wavelength of the SPOT4 satellite	[128]
12	environment	glc_raw3	vegetation derived from the red (0.61-0.68 μm) wavelength of the SPOT4 satellite	[128]
13	environment	bare_4x4	percentage of bare ground (MODIS)	[129]
14	environment	herb_4x4	percentage of herbaceous ground cover (MODIS)	[130]
15	environment	tree_4x4	percentage of woody vegetation (MODIS)	[131]
16	altitude	srtm_c_ln_3x3	elevational contrast calculated from the SRTM30 data set using a 3x3 moving window	[132]
17	altitude	srtm_v_ln_9x9	elevational variance calculated from the SRTM30 data set using a 9x9 moving window	[132]
18	distance	hydro_buf_af	distance to nearest river	[133]

Provided are: number, category assignment, parameter acronym, description of the parameter and the source of the original data.

ENMs were calculated considering 1) all presence data in order to determine the potential distribution of the family Odontobatrachidae in Upper Guinea (OTUcomb) and 2) to those localities assigned to a particular OTU, in order to determine the potential distribution of each molecular lineage. The algorithm and parameters disregard biotic factors limiting species distribution [59]. Consequently, models detected areas that are inaccessible due to geographical immigration barriers or competitive exclusion once a niche is already occupied. Such results should be evaluated with caution when discussing the potential distribution. Details of the jackknifing tests of variable importance to the calculated ENMs are provided in Additional file 14.

### Conservation status

Following IUCN Red List criteria the geographic range, assessed as the Extent of Occurrence (EOO) and the Area of Occupancy (AOO), are among the crucial criteria for classifying a taxon as “Critically Endangered”, “Endangered” or “Vulnerable”. While EOO is roughly defined as the least space contained between all known points and often measured by a minimum convex polygon, AOO refers to the area within the species EOO which is occupied by a taxon (herein OTU) on a 4 km^2^ grid. EOO and AOO were calculated for all recognised OTUs using GeoCAT [126] and according to the IUCN regulation the higher of these two categories is crucial for the final species classification to assess its risk of global extinction [87].

## Availability of supporting data

The data sets supporting the results of this article are included within the article (and its additional files). Gene sequences obtained in the course of this study have been deposited in GenBank under accessions KP005071-KP005450 (see also Additional file 11) and are available in the TreeBASE repository, under http://purl.org/phylo/treebase/phylows/study/TB2:S17293.

## Additional files

Additional file 1:
**Relationships (expanded concatenated tree) and geographical distribution of** ***Odontobatrachus*** **OTUs.**
Additional file 2:
**Number of total clusters/OTUs identified using SpeciesIdentifier at different cut-off thresholds.**
Additional file 3:
**Tree showing results of species delimitation approaches (GMYC and cluster algorithm in SpeciesIdentifier.**
Additional file 4:
**GMYC model results under different tree priors and clock models.**
Additional file 5:
**Dating results of splits between** ***Odontobatrachus*** **OTUs.**
Additional file 6:
**ENM training results of all five OTUs and OTUcomb (OTU1-4 +** ***natator*** **).**
Additional file 7:
**Summary of parameters and their contribution to the ENM approach.**
Additional file 8:
**Hypothetical scenario of dispersal and speciation in the family Odontobatrachidae in the Upper Guinean forest block, West Africa.**
Additional file 9:
**Map of protected areas in Upper Guinean forests, West Africa, and distribution of** ***Odontobatrachus*** **OTUs.**
Additional file 10:
**Mountainous elevations in Upper Guinean forests inhabited by Odontobatrachidae.**
Additional file 11:
**Summary of voucher specimens, and GenBank accession numbers included in the present study.**
Additional file 12:
**Phylogenetic tree of** ***Odontobatrachus*** **OTUs under minimum and maximum partitioning schemes.**
Additional file 13:
**Single gene trees of** ***Odontobatrachus*** **OTUs.**
Additional file 14:
**Results of the jackknifing tests.**

## Abbreviations

*12S*: 12S rRNA
*16S*: 16S rRNA
*cytb*: Cytochrome b
*RAG1*: Recombinase activating protein 1
*SIA*: Seventh-in-absentia
*BDNF*: Brain-derived neurotrophic factor
bp: Base pairs
OTU: Operational taxonomic units
BI: Bayesian inference
ML: Maximum likelihood
BS: Bootstrap support
PP: Posterior probability
BIC: Bayesian information criterion
GTR: General time reversible model
HKY: Hasegawa-Kishino-Yano 85 model
JC: Jukes and Cantor 1969 model
K80: Kimura 1980 model
TIM: Transition model
TPM2uf: Three-parameter model with unequal base frequencies
TrNef: Tamura and Nei 1983 model with equal frequencies
G: Gamma distribution
I: Proportion of invariable sites
ENM: Environmental niche modelling
ROC: Receiver operating characteristic
AUC: Area under the curve
min: Minimum
max: Maximum
SD: Standard deviation
N: Sample size
BM: Natural History Museum, London
MHNG: Natural History Museum, Geneva
ZFMK: Zoologisches Forschungsmuseum Alexander Koenig, Bonn
ZMB: Museum für Naturkunde Berlin
FP: Freetown peninsula population
IL: Inland population
Mt: Mount
IUCN: International union for conservation of nature
RL: Red list
EOO: Extent of occurrence
AOO: Area of occupancy
