# Supplementary material for: Multiple genetic lineages challenge the monospecific status of the West African endemic frog family Odontobatrachidae
Source: BMC Evol Biol. 2015 Apr 19;15:67. doi: 10.1186/s12862-015-0346-9 (PMC4425868; doi:10.1186/s12862-015-0346-9)
Supplement: Additional file 1: — Relationships (expanded concatenated tree) and geographical distribution of Odontobatrachus OTUs. [file 12862_2015_346_MOESM1_ESM.pdf]

1. Relationships (expanded concatenated tree) and geographical distribution of *Odontobatrachus* OTUs

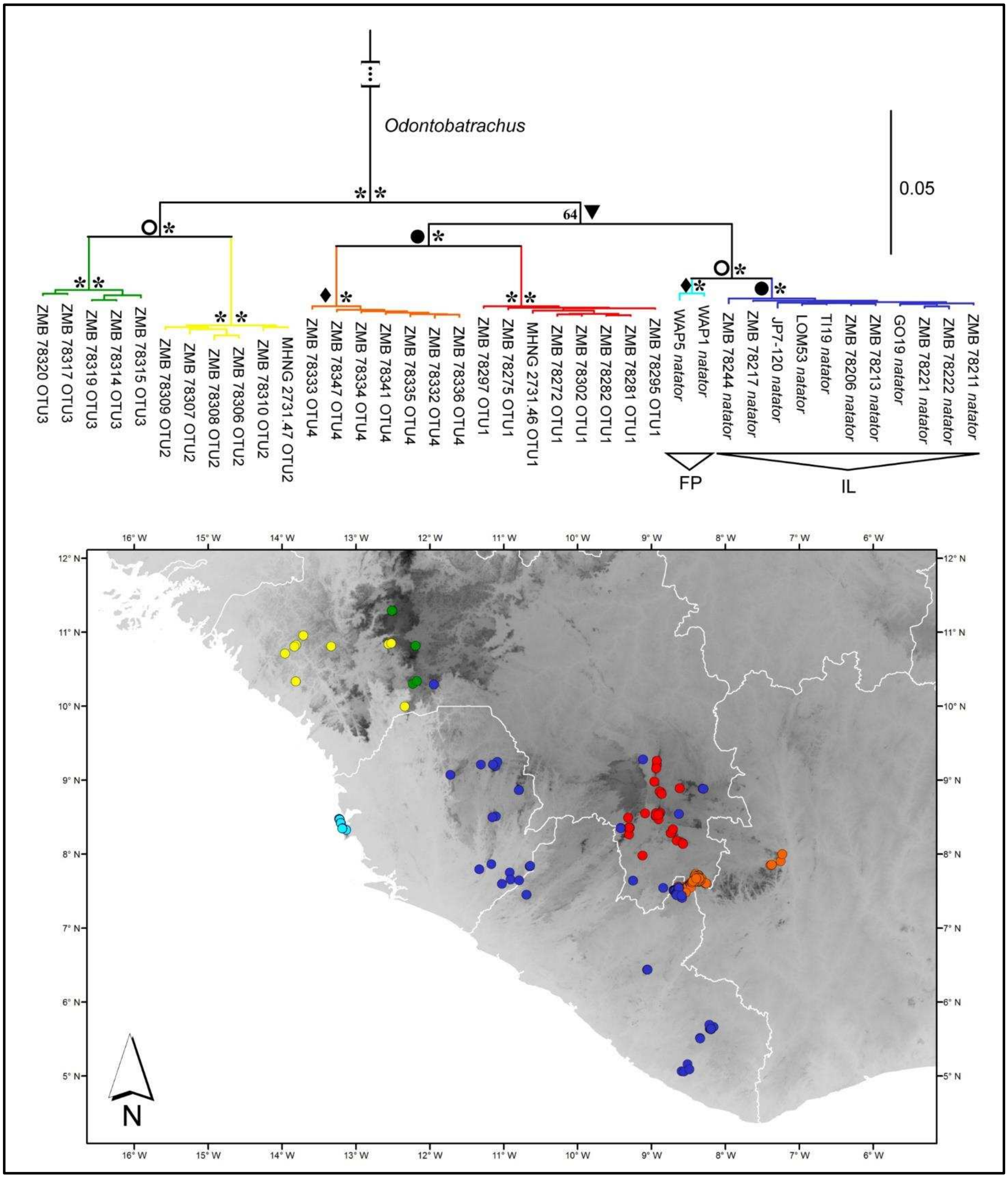

Additional file 1: Relationships (expanded concatenated tree) and geographical distribution of *Odontobatrachus* OTUs. Expanded phylogenetic tree for all vouchers sequenced for all six gene fragments (compare to Figure 1), support values are provided as Bayesian posterior probabilities (right of the branch; PP: * = 1.00; 0.95 ≥ ▼ ≥ 0.99) and ML Boostrap support values (left of the branch; BS: * = 100%; 90 ≥ ♦ ≥ 99; 80 ≥ ● ≥ 89; 70 ≥ ○ ≥ 79). Subclades in *natator* refer to the Freetown Peninsula, Sierra Leone (FP, light blue) and remaining *natator* population further inland (IL, dark blue). Distribution of recognised OTUs is plotted against altitude (altitude increasing with darker colouration).
