# Supplementary material for: Multiple genetic lineages challenge the monospecific status of the West African endemic frog family Odontobatrachidae
Source: BMC Evol Biol. 2015 Apr 19;15:67. doi: 10.1186/s12862-015-0346-9 (PMC4425868; doi:10.1186/s12862-015-0346-9)
Supplement: Additional file 2: — Number of total clusters/OTUs identified using SpeciesIdentifier at different cut-off thresholds. [file 12862_2015_346_MOESM2_ESM.pdf]

Additional file 2: Number of total clusters/OTUs identified using SpeciesIdentifier at different cut-off thresholds. Comparison of the mitochondrial genes *12S* and *16S*. Given are Threshold values (increasing up to a total of 1 OTU), total number of OTUs under a particular threshold, percentage of threshold violation, maximum pairwise distance within recognised OTUs and assignment of recognised OTUs.

Gene	Threshold	Total number of OTUs	Threshold violation (% of total)	Maximum pairwise distance within OTU	recognised OTUs
16S	0.25%	10	5 (50%)	0.5%	---
	0.50%	6	3 (50%)	0.7%	*natator*$_{FP}$, *natator*$_{IL}$, OTU1, OTU2, OTU3, OTU4
	1.0%	6	0 (0%)	0.7%	*natator*$_{FP}$, *natator*$_{IL}$, OTU1, OTU2, OTU3, OTU4
	1.5%	6	0 (0%)	0.7%	*natator*$_{FP}$, *natator*$_{IL}$, OTU1, OTU2, OTU3, OTU4
	2.0%	5	0 (0%)	1.9%	(*natator*$_{FP+IL}$), OTU1, OTU2, OTU3, OTU4
	2.5%	5	0 (0%)	1.9%	(*natator*$_{FP+IL}$), OTU1, OTU2, OTU3, OTU4
	3.0%	4	1 (25%)	3.9%	(*natator*$_{FP+IL}$), (OTU1, OTU4), OTU2, OTU3
	3.5%	3	1 (33%)	5.3%	(*natator*$_{FP+IL}$, OTU1, OTU4), OTU2, OTU3
	4.0%	3	1 (33%)	5.3%	(*natator*$_{FP+IL}$, OTU1, OTU4), OTU2, OTU3
	4.5%	1	1 (100%)	5.8%	(*natator*$_{FP+IL}$, OTU1, OTU2, OTU3, OTU4)
12S	0.50%	10	4 (40%)	1.0%	---
	0.75%	6	4 (67%)	1.3%	*natator*$_{FP}$, *natator*$_{IL}$, OTU1, OTU2, OTU3, OTU4
	1.0%	5	2 (40%)	1.3%	(*natator*$_{FP+IL}$), OTU1, OTU2, OTU3, OTU4
	1.5%	5	0 (0%)	1.3%	(*natator*$_{FP+IL}$), OTU1, OTU2, OTU3, OTU4
	2.0%	5	0 (0%)	1.3%	(*natator*$_{FP+IL}$), OTU1, OTU2, OTU3, OTU4
	2.5%	5	0 (0%)	1.3%	(*natator*$_{FP+IL}$), OTU1, OTU2, OTU3, OTU4
	3.0%	5	0 (0%)	1.3%	(*natator*$_{FP+IL}$), OTU1, OTU2, OTU3, OTU4
	3.5%	4	1 (25%)	4.7%	(*natator*$_{FP+IL}$, OTU4), OTU1, OTU2, OTU3
	4.0%	1	1 (100%)	6.8%	(*natator*$_{FP+IL}$, OTU1, OTU2, OTU3, OTU4)
