# Supplementary material for: Multiple genetic lineages challenge the monospecific status of the West African endemic frog family Odontobatrachidae
Source: BMC Evol Biol. 2015 Apr 19;15:67. doi: 10.1186/s12862-015-0346-9 (PMC4425868; doi:10.1186/s12862-015-0346-9)
Supplement: Additional file 3: — Tree showing results of species delimitation approaches (GMYC and cluster algorithm in SpeciesIdentifier. [file 12862_2015_346_MOESM3_ESM.pdf]

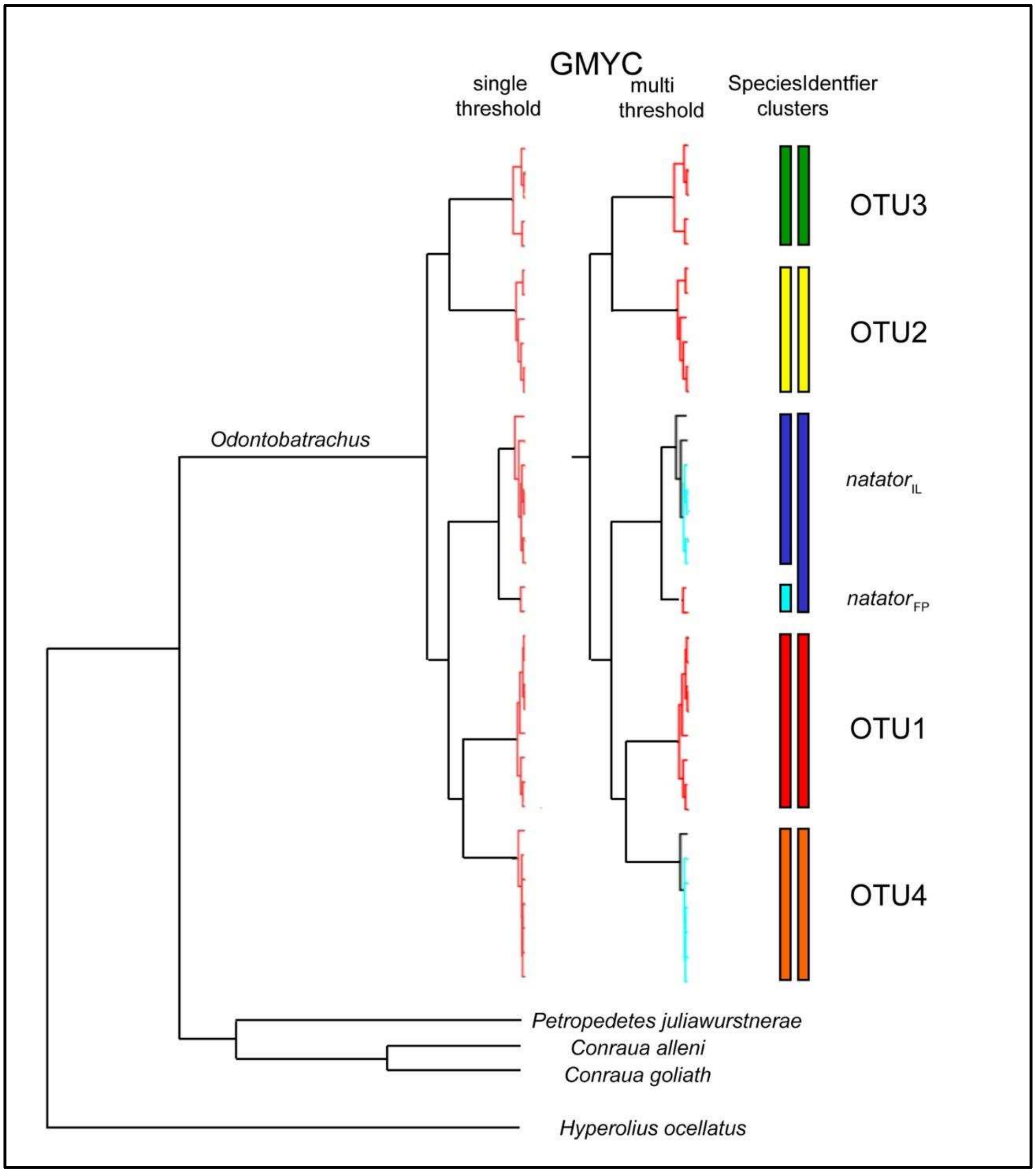

	LR (p)	number of ML clusters	confidence intervals	number of ML entities	confidence intervals
GMYC (single threshold)	4.757989e-05***	6	6 - 8	13	10 - 16
GMYC (multiple threshold)	5.542751e-05***	6	6 - 7	10	10 - 11

Additional file 3: Tree showing results of species delimitation approaches (GMYC and cluster algorithm in SpeciesIdentifier). The full ultrametric tree for West African torrent frogs Odontobatrachidae, *Odontobatrachus*, resulting from 39 taxa (including outgroup taxa) and 3396 bp from six genes, using Bayesian inference analysis with BEAST, a Yule model, and a strict clock. Recognised OTUs are indicated based on three different clustering methods from left to right: 1) GMYC single-threshold (red shading), 2) GMYC multiple-threshold (red shading, blue shading = sub-splits, grey = singletons) and 3) clusters sensu SpeciesIdentifier (shown for comparison). A table summarizing results for this tree additionally provided (LR (p) = likelihood ratio and p value; ML = Maximum likelihood clusters).
