# Supplementary material for: Multiple genetic lineages challenge the monospecific status of the West African endemic frog family Odontobatrachidae
Source: BMC Evol Biol. 2015 Apr 19;15:67. doi: 10.1186/s12862-015-0346-9 (PMC4425868; doi:10.1186/s12862-015-0346-9)
Supplement: Additional file 4: — GMYC model results under different tree priors and clock models. [file 12862_2015_346_MOESM4_ESM.pdf]

4. GMYC model results under different tree priors and clock models.

Additional file 4: GMYC model results under different tree priors and clock models. Shown are the LRT tests for comparison to a null model (likelihood ratio and p value), number of lineages found (clusters + singletons), and the Chi-square test comparing single and multiple GMYC models. The number of independent lineages hypothesized by each run is given under "Total" and refers to Clusters + Singletons.

GMYC model	Tree model	Clock model	LR (p)	Clusters	Singletons	Total	Confidence limit (single/multiple)
Single	Yule	Strict	19.601 (***)	6	4	10	6-7/10-11
Multiple	Yule	Strict	19.906 (***)	6	7	13	6-8/10-16
Single	Yule	Relaxed	9.704 (**)	7	2	9	3-7/3-13
Multiple	Yule	Relaxed	10.769 (**)	7	5	12	5-8/6-19
Single	Coalescent	Strict	17.655(***)	6	4	10	6-6/10-11
Multiple	Coalescent	Strict	20.044 (***)	7	7	14	6-7/10-14
Single	Coalescent	Relaxed	21.528 (***)	6	4	10	6-7/10-11
Multiple	Coalescent	Relaxed	23.262 (***)	7	7	14	6-8/10-16
