# Supplementary material for: Multiple genetic lineages challenge the monospecific status of the West African endemic frog family Odontobatrachidae
Source: BMC Evol Biol. 2015 Apr 19;15:67. doi: 10.1186/s12862-015-0346-9 (PMC4425868; doi:10.1186/s12862-015-0346-9)
Supplement: Additional file 5: — Dating results of splits between Odontobatrachus OTUs. [file 12862_2015_346_MOESM5_ESM.pdf]

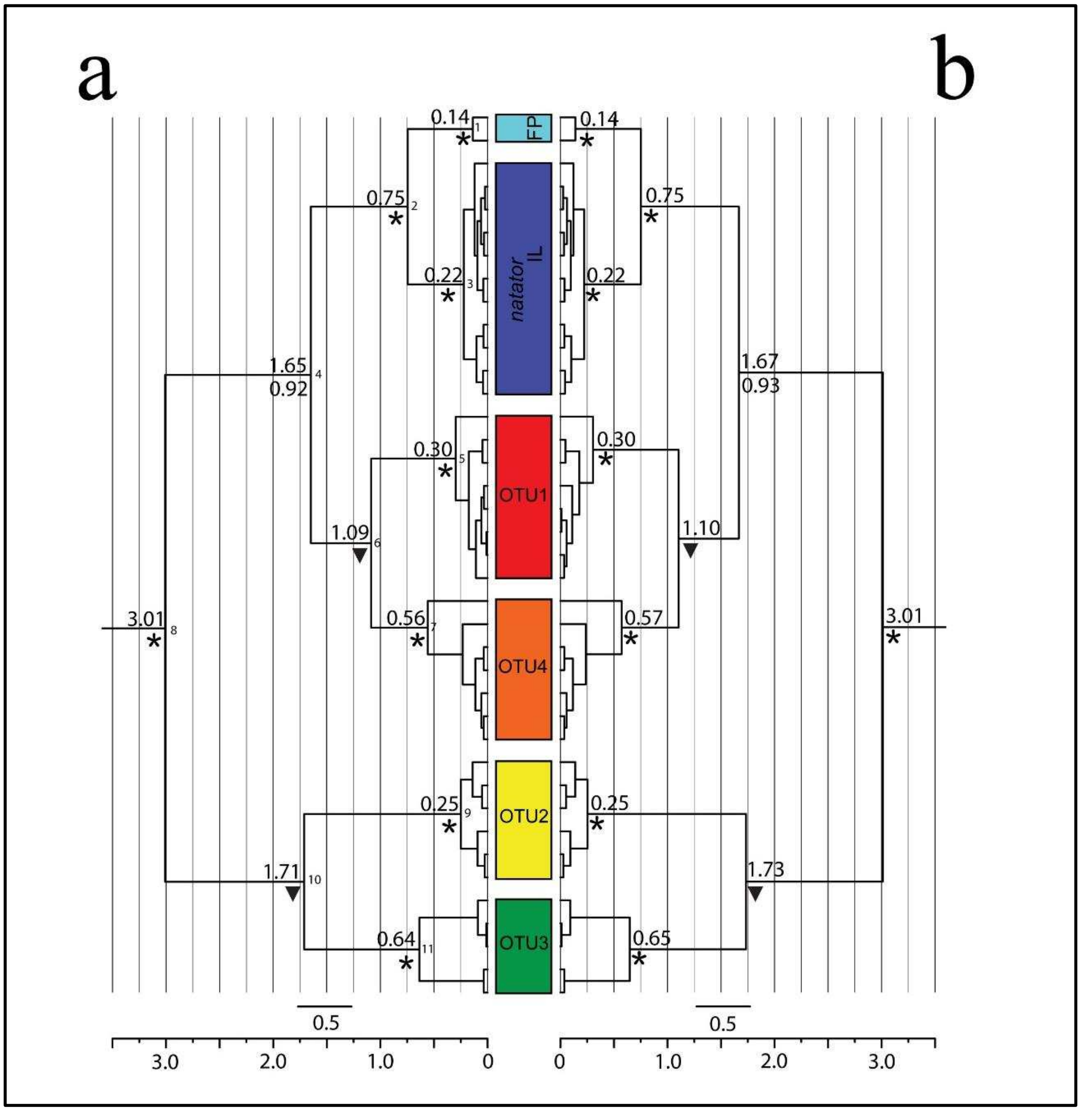

Additional file 5: Dating results of splits between *Odontobatrachus* OTUs. BEAST-trees based on mutation rate estimates for both the mitochondrial genes *12S* + *16S* + *cytb* and the nuclear gene *RAG1* (a) or exclusively for mitochondrial genes *12S* + *16S* + *cytb* (b). Given are mean split ages (above branch) Bayesian posterior probabilities (below branch; PP: * = 1.00; 0.95 ≥ ▼ ≥ 0.99). OTU *natator* is subdivided in two subclades referring to Freetown Peninsula (FP) and remaining inland (IL) populations. The single outgroup *Petropedetes juliawurstnerae* is not shown. The 95% clade intervals of node ages (number right of nodes in left tree) are as follows (mtDNA+nucDNA / mtDNA): 1) 0-1.80Ma / 0-1.70Ma; 2) 0.05-8.86Ma / 0.14-16.45Ma; 3) 0.01-2.73Ma / 0.01-2.44Ma; 4) 0.15-19.19Ma / 0.14-16.45Ma; 5) 0.01-3.71Ma / 0.02-3.41Ma; 6) 0.08-12.62Ma / 0.10-11.32Ma; 7) 0.03-6.85Ma / 0.03-6.27Ma; 8) 0.28-35.07Ma / 0.25-30.70Ma; 9) 0.01-3.02 / 0.01-2.73Ma; 10) 0.13-20.06Ma / 0.11-18.30Ma; 11) 0.02-8.12Ma / 0.01-7.47Ma.
