# Supplementary material for: Multiple genetic lineages challenge the monospecific status of the West African endemic frog family Odontobatrachidae
Source: BMC Evol Biol. 2015 Apr 19;15:67. doi: 10.1186/s12862-015-0346-9 (PMC4425868; doi:10.1186/s12862-015-0346-9)
Supplement: Additional file 6: — ENM training results of all five OTUs and OTUcomb (OTU1-4 + natator ). [file 12862_2015_346_MOESM6_ESM.pdf]

Additional file 6: ENM training results of all five OTUs and OTUcomb (OTU1-4 + *natator*). Provided are number of samples per OTU, regularized and unregularized training gain, number of iterations, training AUC values, and respective test gain and test AUC values.

	#Training samples	Regularized training gain	Unregularized training gain	Iterations	Training AUC	Test gain	Test AUC
natator (average)	55	3.974	4.3526	460.6	0.9958	4.1668	0.9949
OTU1 (average)	27	4.6641	5.2821	501.8	0.9985	5.1188	0.9982
OTU2 (average)	9	3.1082	4.3782	104.4	0.9983	4.3073	0.9982
OTU3 (average)	4	2.2818	2.8088	188.2	0.9956	2.7473	0.9947
OTU4 (average)	44	4.5809	4.9613	496.2	0.9976	4.9645	0.9975
OTU1-4 + *natator* (average)	139	3.5696	3.7477	1560.8	0.9915	3.6507	0.9906
