# Supplementary material for: Multiple genetic lineages challenge the monospecific status of the West African endemic frog family Odontobatrachidae
Source: BMC Evol Biol. 2015 Apr 19;15:67. doi: 10.1186/s12862-015-0346-9 (PMC4425868; doi:10.1186/s12862-015-0346-9)
Supplement: Additional file 7: — Summary of parameters and their contribution to the ENM approach. [file 12862_2015_346_MOESM7_ESM.pdf]

Additional file 7: Summary of parameters and their contribution to the ENM approach. Provided are proportional contribution to the potential distribution in OTU1-4, *natator* and OTUcomb. Highest contributions marked in bold red numbers. Parameter abbreviations as follows: **tmin30_max**: highest value of the minimum temperatures; **tmin30_min**: lowest value of the minimum temperatures; **tmin30_std**: standard deviation of the minimum temperatures; **tmax30_max**: highest value of the maximum temperatures; **tmax30_min**: lowest value of the maximum temperatures; **tmax30_std**: standard deviation the maximum temperatures; **prec30_max**: highest precipitation value (wettest month); **prec30_min**: lowest precipitation value (driest month); **prec30_std**: standard deviation of the precipitation; **prec30_sum**: total annual precipitation; **glc_raw2**: vegetation derived from the near-infrared (0.78-0.89µm) wavelength of the SPOT4 satellite; **glc_raw3**: vegetation derived from the red (0.61-0.68µm) wavelength of the SPOT4 satellite; **bare_4x4**: percentage of bare ground (MODIS); **herb_4x4**: percentage of herbaceous ground cover (MODIS); **tree_4x4**: percentage of woody vegetation (MODIS); **srtm_c_ln_3x3**: elevational contrast calculated from the SRTM30 dataset using a 3x3 moving window; **srtm_v_ln_9x9**: elevational variance calculated from the SRTM30 dataset using a 9x9 moving window; **hydro_buf_af**: distance to nearest river.

	**parameter abbreviation**	***natator***	**OTU1**	**OTU2**	**OTU3**	**OTU4**	**OTUcomb (OTU1-4 + *natator*)**
1	tmin30_max	0.06	0.01	0.00	0.00	0.00	0.98
2	tmin30_min	0.35	0.47	0.00	0.00	0.08	0.53
3	tmin30_std	0.24	12.90	0.00	0.00	8.56	0.97
4	tmax30_max	2.54	0.69	0.00	0.00	1.15	1.88
5	tmax30_min	0.01	1.20	0.00	2.05	0.12	0.66
6	tmax30_std	0.29	0.09	3.19	0.00	2.33	1.39
7	prec30_max	**34.83**	0.26	1.70	0.77	**24.39**	**43.96**
8	prec30_min	16.69	**22.34**	5.67	4.48	**31.00**	2.02
9	prec30_std	0.15	7.88	**49.90**	**46.14**	1.56	0.12
10	prec30_sum	**34.64**	**30.07**	6.24	0.06	1.86	**28.95**
11	glc_raw2	0.01	0.00	0.06	1.81	0.00	0.28
12	glc_raw3	0.22	0.15	0.01	0.00	0.22	0.13
13	bare_4x4	0.29	0.27	**25.75**	**38.12**	1.12	3.89
14	herb_4x4	0.04	0.04	0.00	0.00	0.01	0.58
15	tree_4x4	0.14	0.32	0.00	0.00	0.90	1.50
16	srtm_c_ln_3x3	7.32	10.63	0.35	0.00	1.06	3.70
17	srtm_v_ln_9x9	2.18	12.60	7.05	6.57	**25.63**	8.21
18	hydro_buf_af	0.01	0.07	0.07	0.00	0.00	0.26
