# Supplementary material for: Multiple genetic lineages challenge the monospecific status of the West African endemic frog family Odontobatrachidae
Source: BMC Evol Biol. 2015 Apr 19;15:67. doi: 10.1186/s12862-015-0346-9 (PMC4425868; doi:10.1186/s12862-015-0346-9)
Supplement: Additional file 8: — Hypothetical scenario of dispersal and speciation in the family Odontobatrachidae in the Upper Guinean forest block, West Africa. [file 12862_2015_346_MOESM8_ESM.pdf]

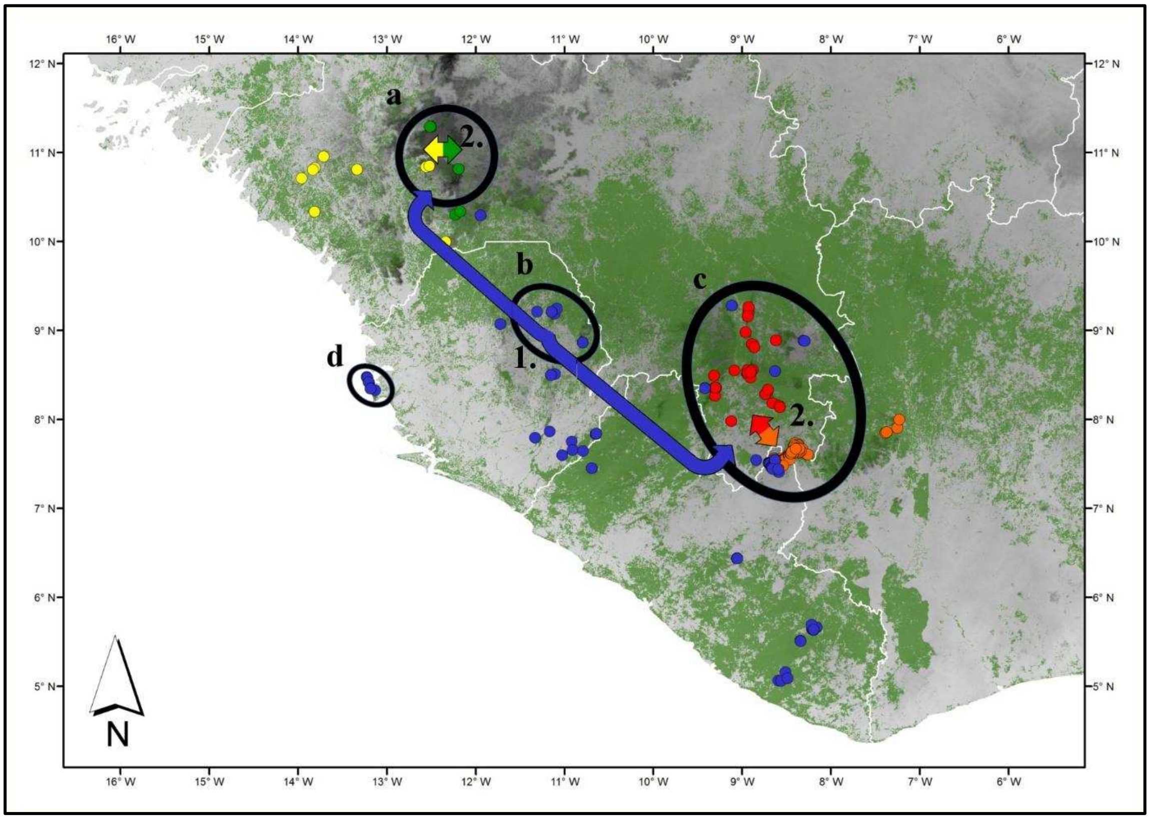

Additional file 8: Hypothetical scenario of dispersal and speciation in the family Odontobatrachidae in the Upper Guinean forest block, West Africa. Arrows refer to dispersal and speciation events (1) Guinea Highlands westwards to the Fouta Djallon and eastwards to Nimba-Simandou Range and (2) within Fouta Djallon and between Nimba Mountains and the Simandou Range. Black circles roughly demarcate supposed refugia: Fouta Djallon (a), Loma Mts.-Tingi Hills (b), Simandou Range-Nimba Mts (c), Peninsula Mountains (d). Arrow colours indicate OTU dispersal; colour code: OTU *natator* = blue; OTU1 = red; OTU2 = yellow; OTU3 = green; OTU4 = orange.
