# Supplementary material for: Multiple genetic lineages challenge the monospecific status of the West African endemic frog family Odontobatrachidae
Source: BMC Evol Biol. 2015 Apr 19;15:67. doi: 10.1186/s12862-015-0346-9 (PMC4425868; doi:10.1186/s12862-015-0346-9)
Supplement: Additional file 9: — Map of protected areas in Upper Guinean forests, West Africa, and distribution of Odontobatrachus OTUs. [file 12862_2015_346_MOESM9_ESM.pdf]

9. Map of protected areas in Upper Guinean forests, West Africa, and distribution of *Odontobatrachus* OTUs

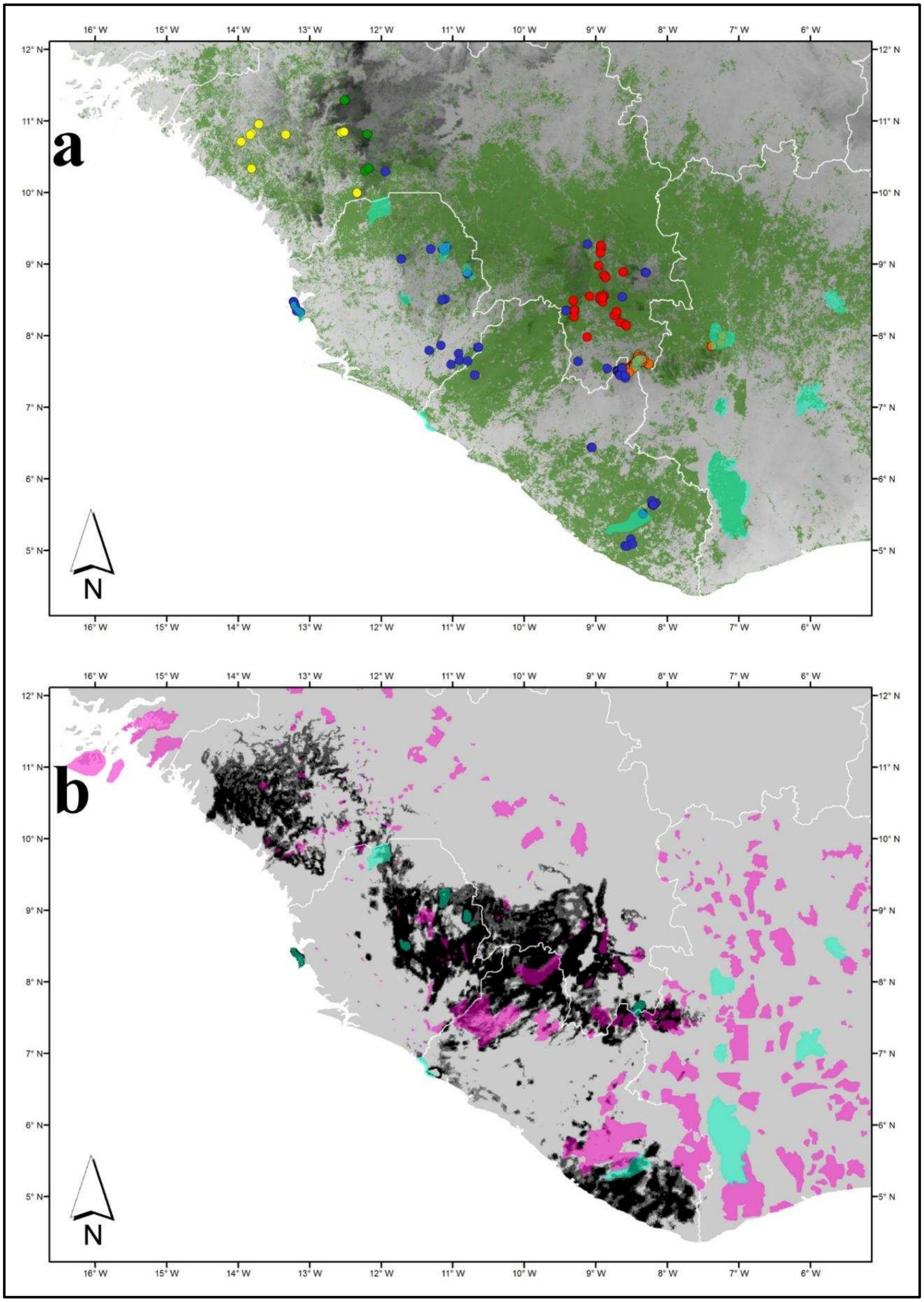

Additional file 9: Map of protected areas in Upper Guinean forests, West Africa, and distribution of *Odontobatrachus* OTUs. Distribution of Odontobatrachidae plotted against National Parks and Biosphere Reserves (turquoise polygons) showing currently protected OTUs; colour code see Figure 2 (a); and Environmental niche modelling (ENM, see Figure 2) map of Odontobatrachidae plotted against National Parks and Biosphere Reserves (turquoise shading) and protected areas of lower priority (Classified Forests, Sanctuaries, etc; pink polygons) showing protected areas potentially comprising Odontobatrachidae OTUs (b).
