# Supplementary material for: Multiple genetic lineages challenge the monospecific status of the West African endemic frog family Odontobatrachidae
Source: BMC Evol Biol. 2015 Apr 19;15:67. doi: 10.1186/s12862-015-0346-9 (PMC4425868; doi:10.1186/s12862-015-0346-9)
Supplement: Additional file 10: — Mountainous elevations in Upper Guinean forests inhabited by Odontobatrachidae. [file 12862_2015_346_MOESM10_ESM.pdf]

10. Mountainous elevations in Upper Guinean forests inhabited by Odontobatrachidae

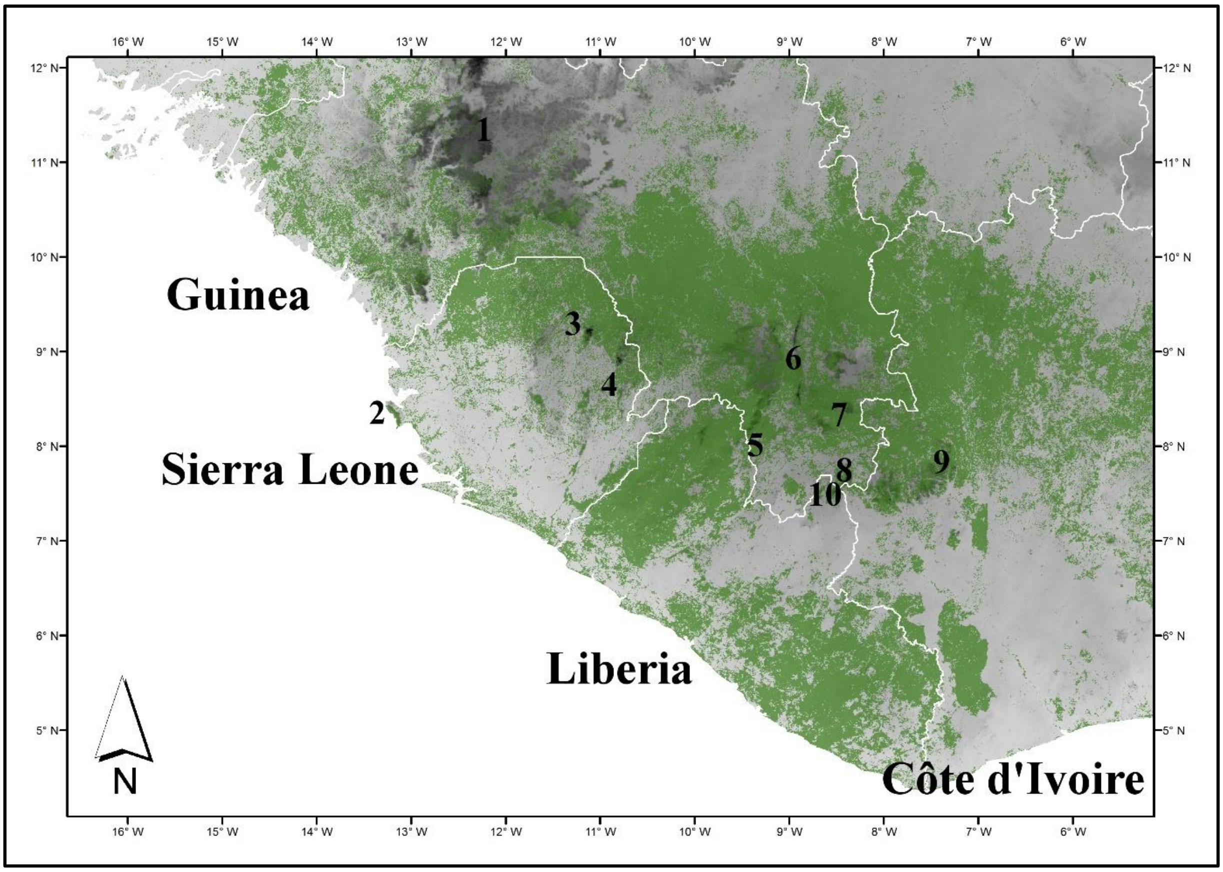

Additional file 10: Mountainous elevations in Upper Guinean forests inhabited by Odontobatrachidae. Indicated region: 1) Fouta Djallon, 2) Peninsula Moutains, 3) Loma Mountains, 4) Tingi Hills, 5) Massif du Ziama, 6) Simandou Range, 7) Mt. Béro, 8) Nimba Mountains, 9) Mt. Sangbé, 10) Mt. Gangra.
