# Supplementary material for: Multiple genetic lineages challenge the monospecific status of the West African endemic frog family Odontobatrachidae
Source: BMC Evol Biol. 2015 Apr 19;15:67. doi: 10.1186/s12862-015-0346-9 (PMC4425868; doi:10.1186/s12862-015-0346-9)
Supplement: Additional file 11: — Summary of voucher specimens, and GenBank accession numbers included in the present study. [file 12862_2015_346_MOESM11_ESM.pdf]

11. Summary of voucher specimens, and GenBank accession numbers included in the present study

Additional file 11: Summary of voucher specimens, and GenBank accession numbers included in the present study. Provided are collection numbers, country affiliation, GenBank numbers for mitochondrial (*12S*, *16S*, *cytb*) and nuclear (*BDNF*, *SIA*, *RAG1*) markers. Sample IDs are as following: The Natural History Museum, London (BM); Natural History Museum, Geneva, Switzerland (MHNG); Zoologisches Forschungsmuseum Alexander Koenig, Bonn, Germany (ZFMK); Museum für Naturkunde Berlin, Germany (ZMB), remaining IDs refer to samples lacking vouchers (CAM, FD, GO, JP7, LOM, MTN, MW, NIM, TI, S01, WAP). Sequences from the Freetown Peninsula are marked as '*natator* (FP)'. Genbank#: AYxxxxxx [1]; JXxxxxxx [2]; KFxxxxxx [3]; new GenBank# KP005071-KP005450). Assignment of vouchers and samples to OTUs following Results section.

Odontobatrachus	Voucher	Region	Country	*16S*	*12S*	*cytb*	*BDNF*	*SIA*	*RAG1*
natator	ZMB 78198	Northern Province	Sierra Leone	KP005071	KP005195				
natator	ZMB 78199	Eastern Province	Sierra Leone	KP005072	KP005196				
natator	ZMB 78200	Northern Province	Sierra Leone	KP005073	KP005197				
natator	ZMB 78206	Eastern Province	Sierra Leone	KF693392	KF693288	KF693672	KF693490	KF693552	KF693612
natator	ZMB 78207	N'Zérékoré Region	Guinea	KP005074	KP005198				
natator	ZMB 78208	N'Zérékoré Region	Guinea	KP005075	KP005199				
natator	ZMB 78209	Kankan Region	Guinea	KP005076	KP005200				
natator	ZMB 78211	N'Zérékoré Region	Guinea	KF693390	KF693286	KF693670	KF693488	KF693550	KF693610
natator	ZMB 78213	N'Zérékoré Region	Guinea	KP005077	KP005201	KP005418	KP005312	KP005377	KP005345
natator	ZMB 78214	N'Zérékoré Region	Guinea	KP005078	KP005202				
natator	ZMB 78217	Mamou Region	Guinea	KP005079	KP005203	KP005419	KP005313	KP005378	KP005346
natator	ZMB 78219	Mamou Region	Guinea	KP005080	KP005204				
natator	ZMB 78220	Grand Cape Mount County	Liberia	KP005081	KP005205				
natator	ZMB 78221	Nimba County	Liberia	KP005082	KP005206	KP005420	KP005314	KP005379	KP005347
natator	ZMB 78222	Nimba County	Liberia	KP005083	KP005207	KP005421	KP005315	KP005380	KP005348
natator	ZMB 78224	Nimba County	Liberia	KP005084	KP005208			KP005381	
natator	ZMB 78225	Nimba County	Liberia	KP005085	KP005209			KP005382	
natator	ZMB 78244	Grand Gedeh County	Liberia	KF693391	KF693286	KF693670	KF693488	KF693550	KF693610
natator	ZMB 78246	Grand Gedeh County	Liberia	KP005086	KP005210			KP005383	
natator	ZMB 78247	Grand Gedeh County	Liberia	KP005087	KP005211			KP005384	
natator	ZMB 78248	Grand Gedeh County	Liberia	KP005088	KP005212			KP005385	
natator	ZMB 78303	N'Zérékoré Region	Guinea	KP005089	---				
natator	ZMB 80500	Sinoe County	Liberia	KP005090	---				
natator	ZMB 80501	Sinoe County	Liberia	KP005091	---				
natator	ZMB 80502	Sinoe County	Liberia	KP005092	---				
natator	ZMB 80503	Sinoe County	Liberia	KP005093	---				
natator	GO15	Grand Cape Mount County	Liberia	KP005094	KP005213				
natator	GO19	Grand Cape Mount County	Liberia	KP005095	KP005214	KP005422	KP005316	KP005386	KP005349
natator	JP7-119	N'Zérékoré Region	Guinea	KP005096	KP005215				
natator	JP7-120	N'Zérékoré Region	Guinea	KP005097	KP005216	KP005423	KP005317	KP005387	KP005350
natator	LOM53	Northern Province	Sierra Leone	KP005098	KP005217	KP005424	KP005318	KP005388	KP005351
natator	Nim96	Eastern Province	Sierra Leone	KP005099	KP005218				
natator	TI18	Eastern Province	Sierra Leone	KP005100	KP005219				
natator	TI19	Eastern Province	Sierra Leone	KP005101	KP005220	KP005425	KP005319	KP005389	KP005352
natator	MW6025	Northern Province	Sierra Leone	JX546953	JX546938	JX546968			
natator	BM 2005.1382	Northern Province	Sierra Leone	JX546954	JX546939	JX546969			
natator (FP)	WAP1	Western Area	Sierra Leone	KP005102	KP005221	KP005426	KP005320	KP005390	KP005353
natator (FP)	WAP5	Western Area	Sierra Leone	KP005103	KP005222	KP005427	KP005321	KP005391	KP005354
OTU1	MHNG 2731.45	N'Zérékoré Region	Guinea	KP005104	KP005223				
OTU1	MHNG 2731.46	N'Zérékoré Region	Guinea	KP005105	KP005224	KP005428	KP005322	KP005392	KP005355
OTU1	ZFMK 95465	N'Zérékoré Region	Guinea	KP005106	KP005225				
OTU1	ZMB 78251	Kankan Region	Guinea	KP005107	KP005226				
OTU1	ZMB 78256	N'Zérékoré Region	Guinea	KP005108	KP005227				
OTU1	ZMB 78259	Kankan Region	Guinea	KP005109	KP005228				
OTU1	ZMB 78261	Kankan Region	Guinea	KP005110	KP005229				
OTU1	ZMB 78262	Kankan Region	Guinea	KP005111	KP005230				
OTU1	ZMB 78266	Kankan Region	Guinea	KP005112	KP005231				
OTU1	ZMB 78269	Kankan Region	Guinea	KP005113	KP005232				
OTU1	ZMB 78271	N'Zérékoré Region	Guinea	KP005114	KP005233				
OTU1	ZMB 78272	Kankan Region	Guinea	KP005115	KP005234	KP005429	KP005323	KP005393	KP005356
OTU1	ZMB 78273	N'Zérékoré Region	Guinea	KP005116	KP005235				
OTU1	ZMB 78274	N'Zérékoré Region	Guinea	KP005117	KP005236				
OTU1	ZMB 78275	N'Zérékoré Region	Guinea	KP005118	KP005237	KP005430	KP005324	KP005394	KP005357
OTU1	ZMB 78276	N'Zérékoré Region	Guinea	KP005119	KP005238				
OTU1	ZMB 78277	N'Zérékoré Region	Guinea	KP005120	KP005239				
OTU1	ZMB 78278	N'Zérékoré Region	Guinea	KP005121	KP005240				
OTU1	ZMB 78280	N'Zérékoré Region	Guinea	KP005122	KP005241				
OTU1	ZMB 78281	N'Zérékoré Region	Guinea	KP005123	KP005242	KP005431	KP005325	KP005395	KP005358
OTU1	ZMB 78282	N'Zérékoré Region	Guinea	KP005124	KP005243	KP005432	KP005326	KP005396	KP005359

11. Summary of voucher specimens, and GenBank accession numbers included in the present study

Additional file 11 (continued).

Odontobatrachus	Voucher	Region	Country	*16S*	*12S*	*cytb*	*BDNF*	*SIA*	*RAG1*
OTU1	ZMB 78283	N’Zérékoré Region	Guinea	KP005125	---				
OTU1	ZMB 78284	N’Zérékoré Region	Guinea	KP005126	KP005244				
OTU1	ZMB 78285	N’Zérékoré Region	Guinea	KP005127	KP005245				
OTU1	ZMB 78286	N’Zérékoré Region	Guinea	KP005128	---				
OTU1	ZMB 78289	N’Zérékoré Region	Guinea	KP005129	KP005246				
OTU1	ZMB 78290	N’Zérékoré Region	Guinea	KP005130	KP005247				
OTU1	ZMB 78295	N’Zérékoré Region	Guinea	KP005131	KP005248	KP005433	KP005327	KP005397	KP005360
OTU1	ZMB 78297	N’Zérékoré Region	Guinea	KP005132	KP005249	KP005434	KP005328	KP005398	KP005361
OTU1	ZMB 78299	N’Zérékoré Region	Guinea	KP005133	KP005250				
OTU1	ZMB 78300	N’Zérékoré Region	Guinea	KP005134	KP005251				
OTU1	ZMB 78302	N’Zérékoré Region	Guinea	KP005135	KP005252	KP005435	KP005329	KP005399	KP005362
OTU2	MHNG 2731.47	Mamou Region	Guinea	KP005136	KP005253	KP005436	KP005330	KP005400	KP005363
OTU2	ZFMK 95466	Kindia Region	Guinea	KP005137	KP005254				
OTU2	ZMB 78305	Kindia Region	Guinea	KP005138	KP005255				
OTU2	ZMB 78306	Kindia Region	Guinea	KP005139	KP005256	KP005437	KP005331	KP005401	KP005364
OTU2	ZMB 78307	Kindia Region	Guinea	KP005140	KP005257	KP005438	KP005332	KP005402	KP005365
OTU2	ZMB 78308	Kindia Region	Guinea	KP005141	KP005258	KP005439	KP005333	KP005403	KP005366
OTU2	ZMB 78309	Kindia Region	Guinea	KP005142	KP005259	KP005440	KP005334	KP005404	KP005367
OTU2	ZMB 78310	Mamou Region	Guinea	KP005143	KP005260	KP005441	KP005335	KP005405	KP005368
OTU2	ZMB 78311	Mamou Region	Guinea	KP005144	KP005261				
OTU2	ZMB 78313	Mamou Region	Guinea	KP005145	KP005262				
OTU3	MHNG 2731.48	Labé Region	Guinea	KP005146	KP005263			KP005406	
OTU3	ZMB 78314	Labé Region	Guinea	KP005147	KP005264	KP005442	KP005336	KP005407	KP005369
OTU3	ZMB 78315	Labé Region	Guinea	KF693393	KF693289	KF693673	KF693491	KF693553	KF693613
OTU3	ZMB 78316	Labé Region	Guinea	KP005148	KP005265			KP005408	
OTU3	ZMB 78317	Mamou Region	Guinea	KF693394	KF693290	KF693674	KF693492	KF693554	KF693614
OTU3	ZMB 78318	Mamou Region	Guinea	KP005149	KP005266			KP005409	
OTU3	ZMB 78319	Labé Region	Guinea	KF693395	KF693291	KF693675	KF693493	KF693555	KF693615
OTU3	ZMB 78320	Mamou Region	Guinea	KP005150	KP005267	KP005443	KP005337	KP005410	KP005370
OTU3	ZMB 78323	Mamou Region	Guinea	KP005151	KP005268				
OTU3	FD1	Labé Region	Guinea	KP005152	KP005269				
OTU3	FD2	Labé Region	Guinea	KP005153	KP005270				
OTU4	MHNG 2731.49	N’Zérékoré Region	Guinea	KP005154	---				
OTU4	MHNG 2731.50	N’Zérékoré Region	Guinea	KP005155	KP005271				
OTU4	ZFMK 95467	N’Zérékoré Region	Guinea	KP005156	KP005272				
OTU4	ZFMK 95468	N’Zérékoré Region	Guinea	KP005157	KP005273				
OTU4	ZMB 78332	Nimba County	Liberia	KP005158	KP005274	KP005444	KP005338	KP005411	KP005371
OTU4	ZMB 78333	Nimba County	Liberia	KP005159	KP005275	KP005445	KP005339	KP005412	---
OTU4	ZMB 78334	Nimba County	Liberia	KP005160	KP005276	KP005446	KP005340	KP005413	KP005372
OTU4	ZMB 78335	Nimba County	Liberia	KP005161	KP005277	KP005447	KP005341	KP005414	KP005373
OTU4	ZMB 78336	N’Zérékoré Region	Guinea	KP005162	KP005278	KP005448	KP005342	KP005415	KP005374
OTU4	ZMB 78337	N’Zérékoré Region	Guinea	KP005163	KP005279				
OTU4	ZMB 78340	N’Zérékoré Region	Guinea	KP005164	KP005280				
OTU4	ZMB 78341	N’Zérékoré Region	Guinea	KP005165	KP005281	KP005449	KP005343	KP005416	KP005375
OTU4	ZMB 78347	N’Zérékoré Region	Guinea	KP005166	KP005282	KP005450	KP005344	KP005417	KP005376
OTU4	ZMB 78348	N’Zérékoré Region	Guinea	KP005167	KP005283				
OTU4	ZMB 78349	N’Zérékoré Region	Guinea	KP005168	KP005284				
OTU4	ZMB 78350	N’Zérékoré Region	Guinea	KP005169	KP005285				
OTU4	ZMB 78352	N’Zérékoré Region	Guinea	KP005170	KP005286				
OTU4	ZMB 78355	N’Zérékoré Region	Guinea	KP005171	KP005287				
OTU4	ZMB 78356	N’Zérékoré Region	Guinea	KP005172	---				
OTU4	ZMB 78357	N’Zérékoré Region	Guinea	KP005173	KP005288				
OTU4	ZMB 78358	N’Zérékoré Region	Guinea	KP005174	KP005289				
OTU4	ZMB 78359	N’Zérékoré Region	Guinea	KP005175	KP005290				
OTU4	ZMB 78360	N’Zérékoré Region	Guinea	KP005176	KP005291				
OTU4	ZMB 78362	N’Zérékoré Region	Guinea	KP005177	KP005292				
OTU4	ZMB 78363	N’Zérékoré Region	Guinea	KP005178	KP005293				
OTU4	ZMB 78364	N’Zérékoré Region	Guinea	KP005179	KP005294				
OTU4	ZMB 78366	N’Zérékoré Region	Guinea	KP005180	KP005295				
OTU4	ZMB 78368	N’Zérékoré Region	Guinea	KP005181	KP005296				
OTU4	ZMB 78369	N’Zérékoré Region	Guinea	KP005182	KP005297				
OTU4	ZMB 78370	N’Zérékoré Region	Guinea	KP005183	KP005298				
OTU4	ZMB 78371	N’Zérékoré Region	Guinea	KP005184	KP005299				
OTU4	ZMB 78372	N’Zérékoré Region	Guinea	KP005185	KP005300				
OTU4	ZMB 78373	N’Zérékoré Region	Guinea	KP005186	KP005301				
OTU4	ZMB 78374	N’Zérékoré Region	Guinea	KP005187	KP005302				
OTU4	ZMB 78375	N’Zérékoré Region	Guinea	---	KP005303				
OTU4	ZMB 78376	N’Zérékoré Region	Guinea	KP005188	KP005304				
OTU4	ZMB 78377	N’Zérékoré Region	Guinea	KP005189	KP005305				

Additional file 11 (continued).

Odontobatrachus	Voucher	Region	Country	*16S*	*12S*	*cytb*	*BDNF*	*SIA*	*RAG1*
OTU4	ZMB 78378	N'Zérékoré Region	Guinea	---	KP005306				
OTU4	ZMB 78379	N'Zérékoré Region	Guinea	KP005190	KP005307				
OTU4	ZMB 78380	N'Zérékoré Region	Guinea	KP005191	KP005308				
OTU4	ZMB78381	N'Zérékoré Region	Guinea	KP005192	KP005309				
OTU4	MTN1	N'Zérékoré Region	Guinea	KP005193	KP005310				
OTU4	MTN2	N'Zérékoré Region	Guinea	KP005194	KP005311				
OTU4	S01.58	Dix-Huit Montagnes Region	Ivory Coast	AY902379	---				
Hyperolius ocellatus	MHNG 2715.58		Cameroon	KF693379	KF693275	KF693659	KF693477	KF693539	KF693599
Conraua goliath	CAM1		Cameroon	KF693384	KF693280	KF693664	KF693482	KF693544	KF693604
Conraua alleni	ZMB 78428		Liberia	KF693387	KF693283	KF693667	KF693485	KF693547	KF693607
Petropedetes juliawurstnerae	MHNG 2713.19		Cameroon	KF693419	KF693323	KF693693	KF693517	KF693578	KF693637

1. Rödel M-O, Kosuch J, Kouamé NG, Ernst R, Veith M: ***Phrynobatrachus alticola* Guibé & Lamotte, 1961 is a junior synonym of *Phrynobatrachus tokba* (Chabanaud, 1921).** *Afric J Herpetol* 2005, **54**:93-98.
2. Loader SP, Ceccarelli FS, Wilkinson M, Menegon M, de Mariaux J, de Sá RO, Howell KM, Gower DJ: **Species boundaries and biogeography of East African torrent frogs of the genus *Petropedetes* (Amphibia: Anura: Petropeditidae).** *Afric J Herpetol* 2013, **62**:40-48.
3. Barej MF, Rödel M-O, Loader SP, Menegon M, Gonwouo NL, Penner J, Gvoždík V, Bell RC, Nagel P, Schmitz A**: Light shines through the spindrift – phylogeny of African Torrent Frogs (Amphibia, Anura, Petropedetidae).** *Mol Phyl Evol* 2014 **71**:261-273.
