# Supplementary material for: Multiple genetic lineages challenge the monospecific status of the West African endemic frog family Odontobatrachidae
Source: BMC Evol Biol. 2015 Apr 19;15:67. doi: 10.1186/s12862-015-0346-9 (PMC4425868; doi:10.1186/s12862-015-0346-9)
Supplement: Additional file 12: — Phylogenetic tree of Odontobatrachus OTUs under minimum and maximum partitioning schemes. [file 12862_2015_346_MOESM12_ESM.pdf]

12. Phylogenetic tree of *Odontobatrachus* OTUs under minimum and maximum partitioning schemes

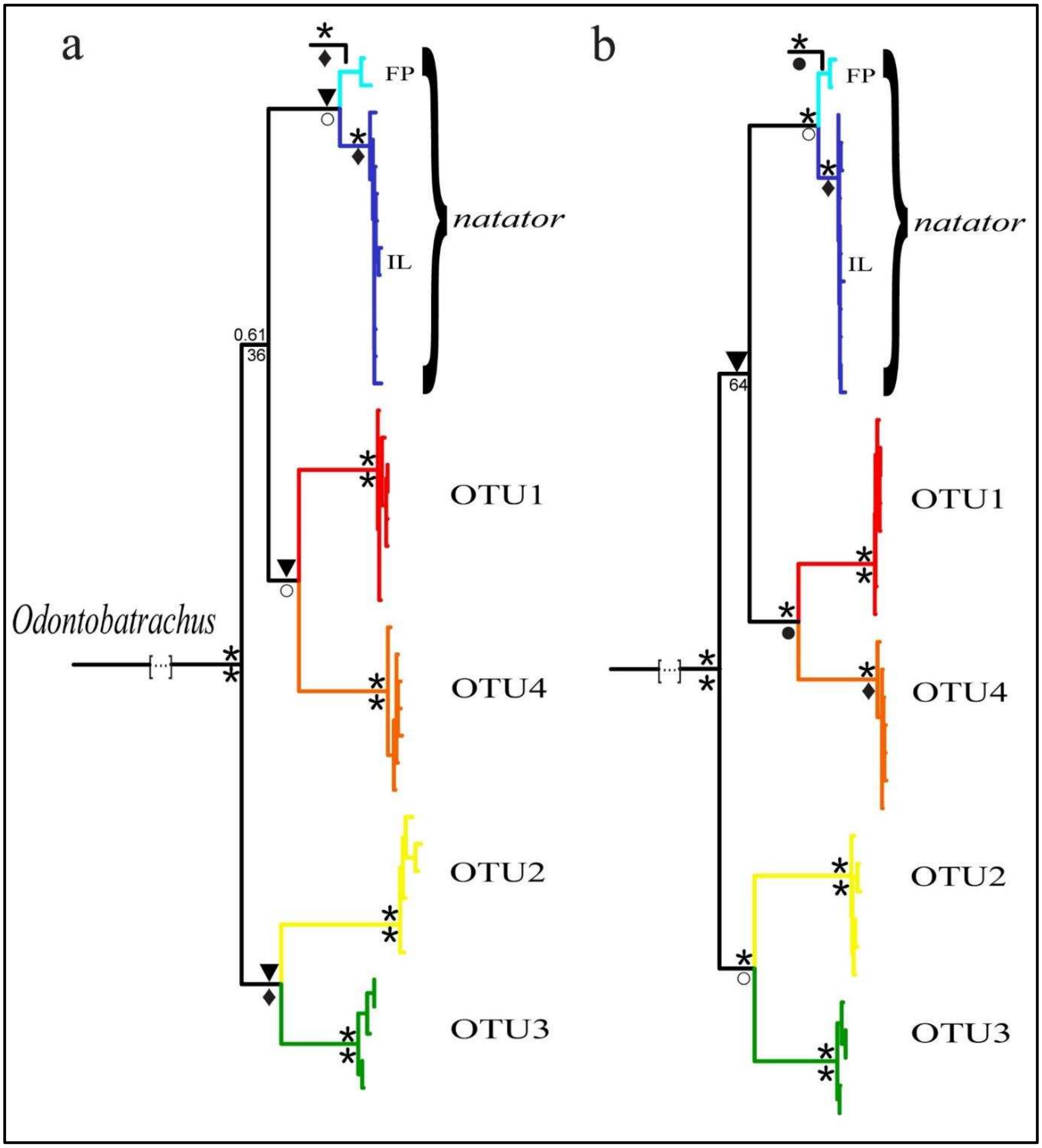

Additional file 12: Phylogenetic tree of *Odontobatrachus* OTUs under minimum and maximum partitioning schemes. Trees resulting from minimum and maximum partitioned Bayes and ML analyses (a: unpartitioned; b: 14 partitions) of mitochondrial genes *16S*, *12S*, *cytb* and nuclear genes *RAG1*, *SIA* and *BDNF* showing differences in the support level according to partition scheme (outgroups not shown). Support values are provided as Bayesian posterior probabilities (above branch; PP: * = 1.00; 0.95 ≥ ▼ ≥ 0.99) and Boostrap support values (below branch; BS: * = 100%; 90 ≥ ♦ ≥ 99; 80 ≥ ● ≥ 89; 70 ≥ ○ ≥ 79). OTU *natator* is subdivided in two subclades referring to Freetown Peninsula (FP) and remaining inland (IL) populations.
