# Supplementary material for: Multiple genetic lineages challenge the monospecific status of the West African endemic frog family Odontobatrachidae
Source: BMC Evol Biol. 2015 Apr 19;15:67. doi: 10.1186/s12862-015-0346-9 (PMC4425868; doi:10.1186/s12862-015-0346-9)
Supplement: Additional file 13: — Single gene trees of Odontobatrachus OTUs. [file 12862_2015_346_MOESM13_ESM.pdf]

Additional file 13: Single gene trees of *Odontobatrachus* OTUs. Trees resulting from Maximum Likelihood (left) and Bayesian Inference (right) of mitochondrial genes *16S* (Additional file 13.1), *12S* (Additional file 13.2), *cytb* (Additional file 13.3) and nuclear genes *RAG1* (Additional file 13.4), *SIA* (Additional file 13.5) and *BDNF* (Additional file 13.6), outgroups (N= 4) and OTU internal node-values not shown.

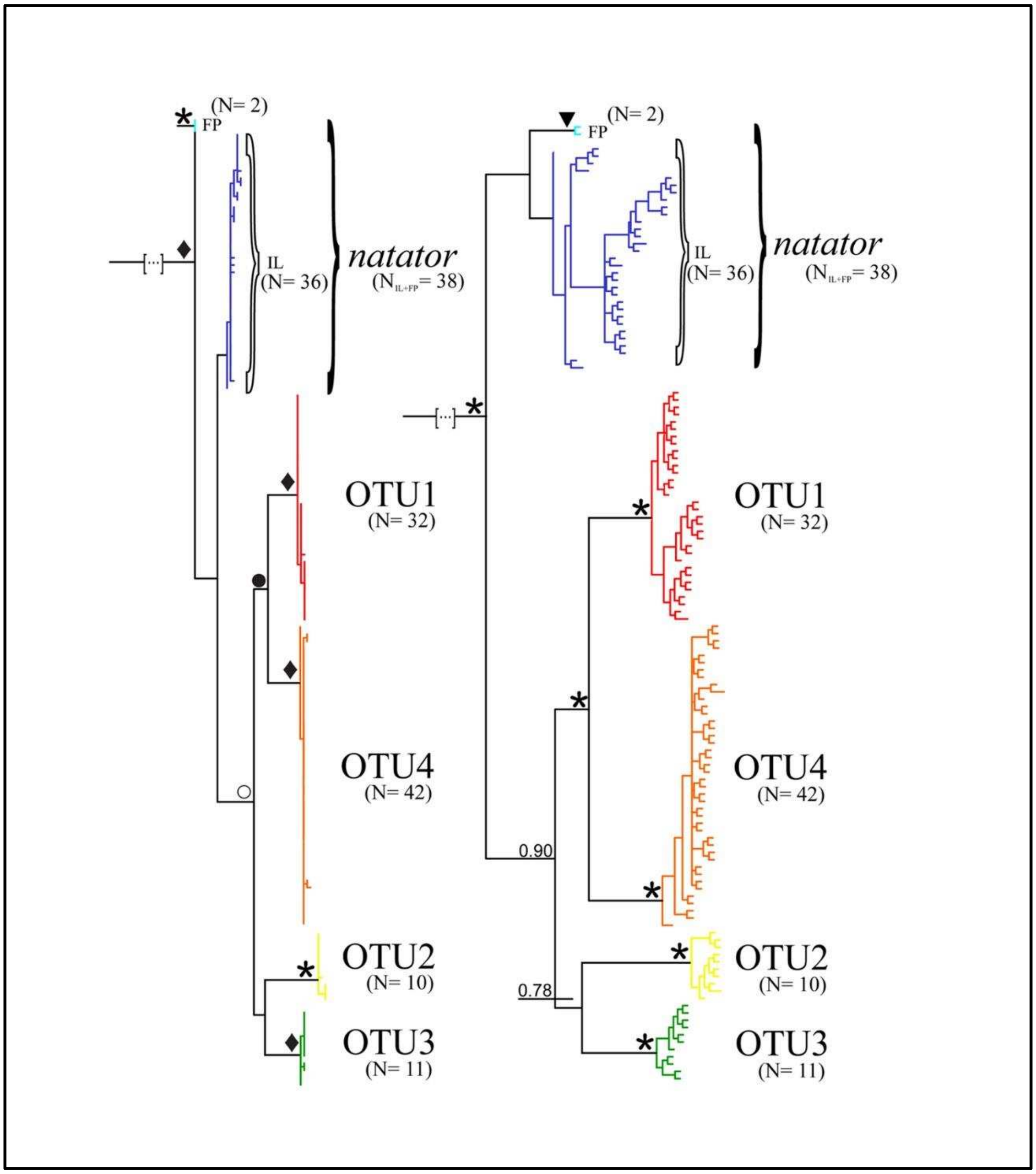

Additional file 13.1: Single gene trees of *Odontobatrachus* OTUs based on the mitochondrial *16S* gene. Trees resulting from Maximum Likelihood (left) and Bayesian Inference (right), outgroups not shown. Support values are provided as Bayesian posterior probabilities (PP: * = 1.00; 0.95 ≥ ▼ ≥ 0.99; PP values below 0.70 not shown) and Boostrap support values (BS: * = 100%; 90 ≥ ♦ ≥ 99; 80 ≥ ● ≥ 89; 70 ≥ ○ ≥ 79; BS values below 50 not shown). OTU *natator* is subdivided in two subclades referring to Freetown Peninsula (FP) and remaining inland (IL) populations.

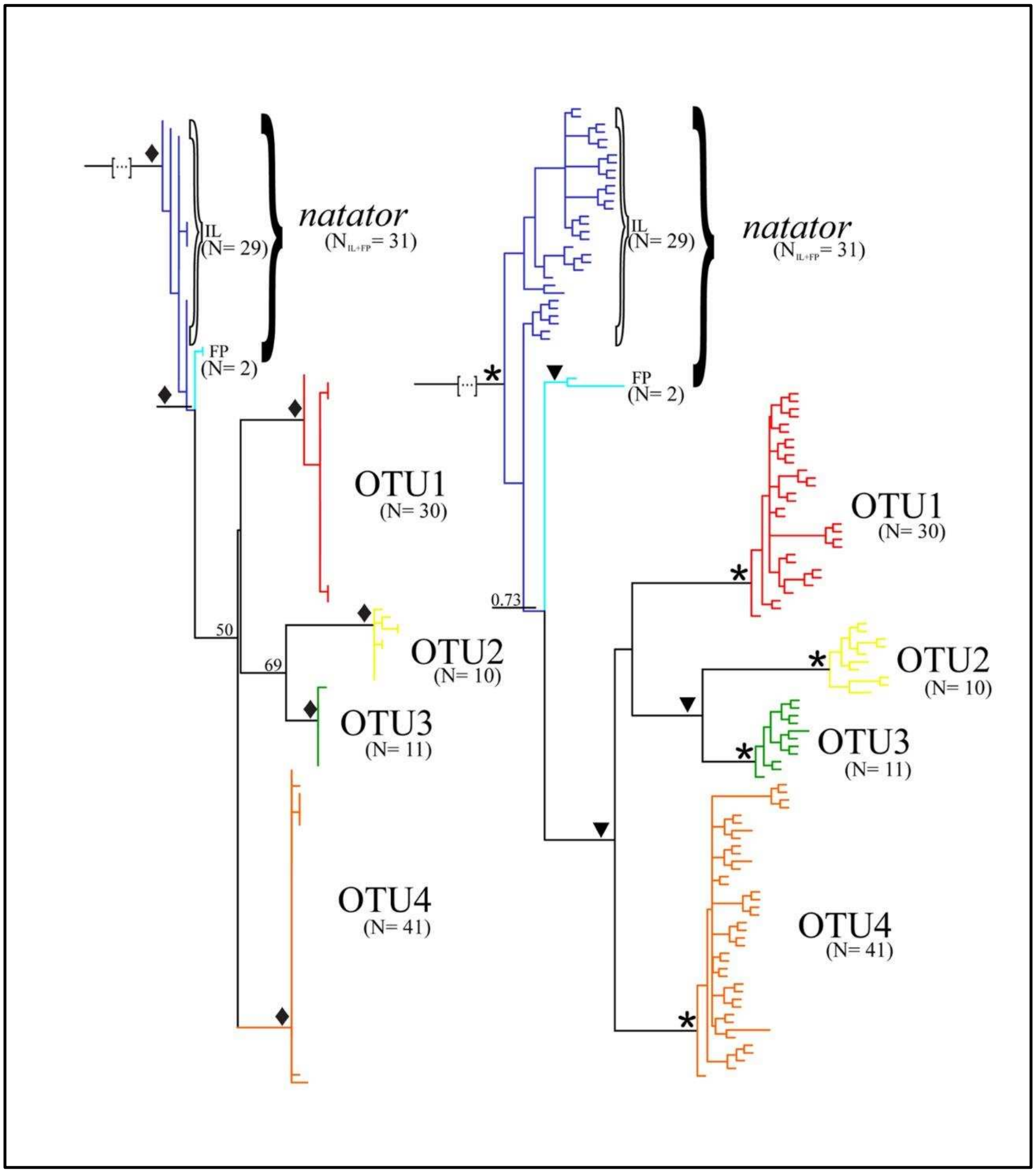

Additional file 13.2: Single gene trees of *Odontobatrachus* OTUs based on the mitochondrial *12S* gene. Trees resulting from Maximum Likelihood (left) and Bayesian Inference (right), outgroups not shown. Support values are provided as Bayesian posterior probabilities (PP: * = 1.00; 0.95 ≥ ▼ ≥ 0.99; PP values below 0.70 not shown) and Boostrap support values (BS: * = 100%; 90 ≥ ♦ ≥ 99; 80 ≥ ● ≥ 89; 70 ≥ ○ ≥ 79; BS values below 50 not shown). OTU *natator* is subdivided in two subclades referring to Freetown Peninsula (FP) and remaining inland (IL) populations.

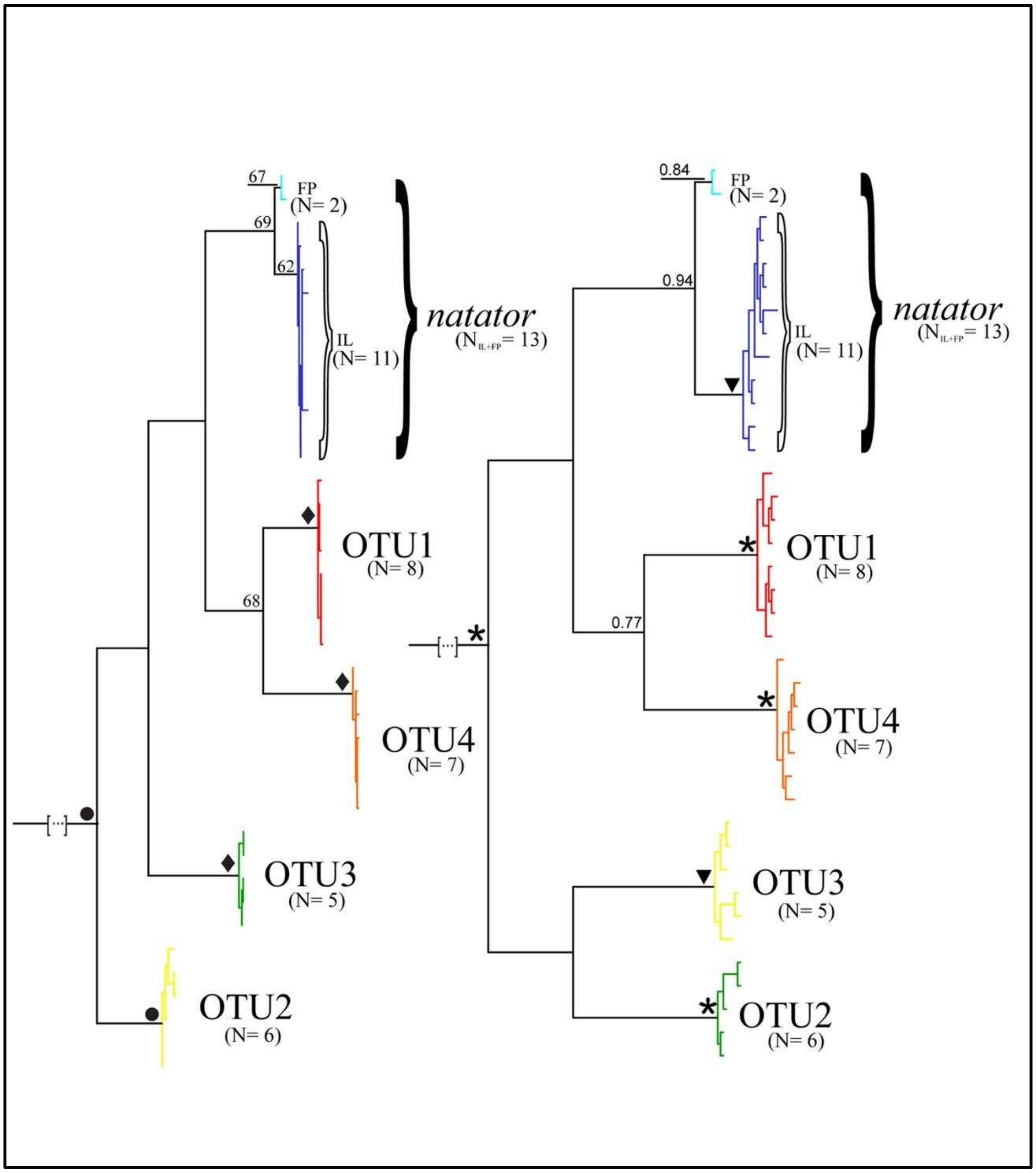

Additional file 13.3: Single gene trees of *Odontobatrachus* OTUs based on the mitochondrial *cytb* gene. Trees resulting from Maximum Likelihood (left) and Bayesian Inference (right), outgroups not shown. Support values are provided as Bayesian posterior probabilities (PP: * = 1.00; 0.95 ≥ ▼ ≥ 0.99; PP values below 0.70 not shown) and Boostrap support values (BS: * = 100%; 90 ≥ ♦ ≥ 99; 80 ≥ ● ≥ 89; 70 ≥ ○ ≥ 79; BS values below 50 not shown). OTU *natator* is subdivided in two subclades referring to Freetown Peninsula (FP) and remaining inland (IL) populations.

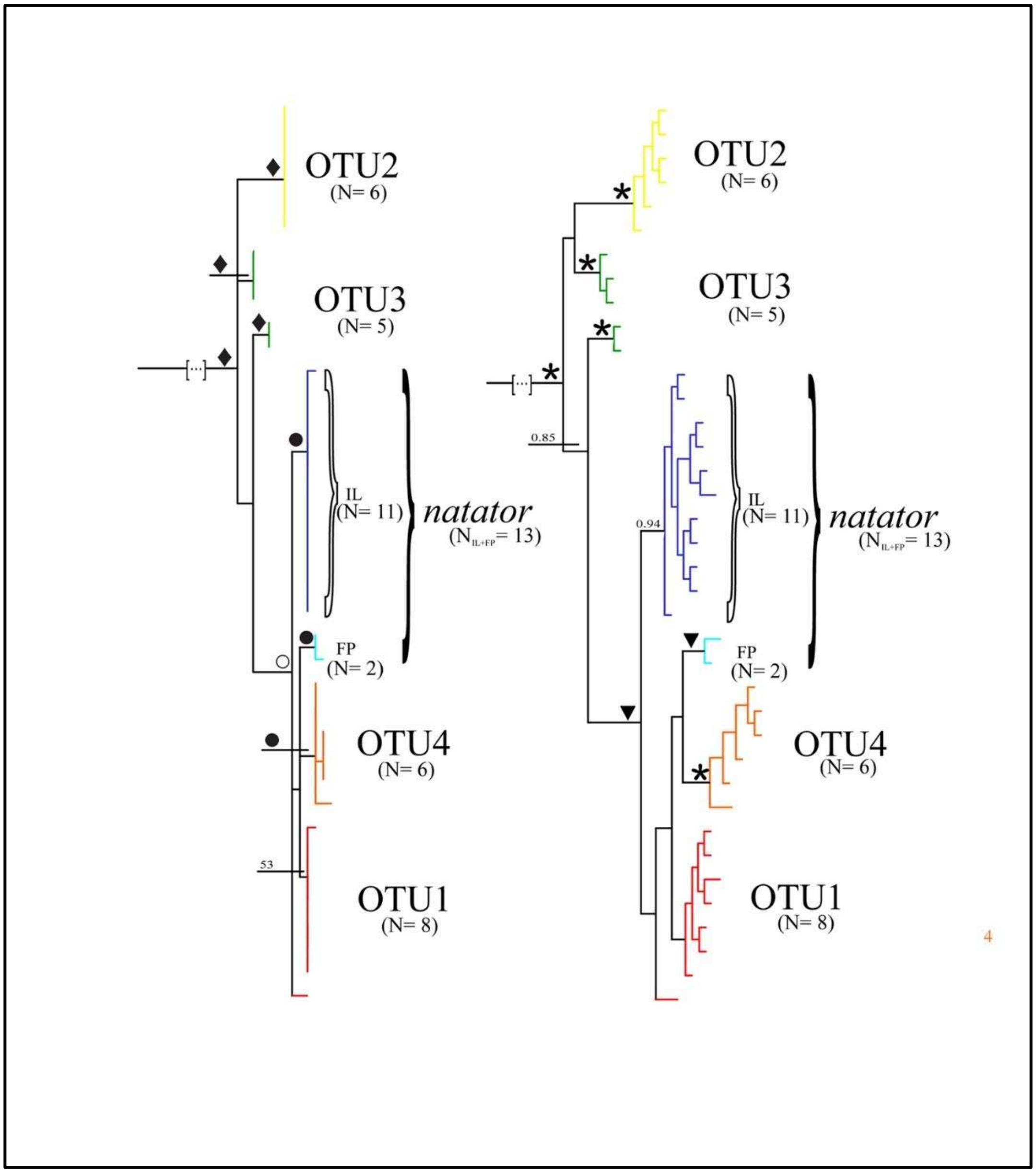

Additional file 13.4: Single gene trees of *Odontobatrachus* OTUs based on the nuclear *RAG1* gene. Trees resulting from Maximum Likelihood (left) and Bayesian Inference (right), outgroups not shown. Support values are provided as Bayesian posterior probabilities (PP: * = 1.00; 0.95 ≥ ▼ ≥ 0.99; PP values below 0.70 not shown) and Boostrap support values (BS: * = 100%; 90 ≥ ♦ ≥ 99; 80 ≥ ● ≥ 89; 70 ≥ ○ ≥ 79; BS values below 50 not shown). OTU *natator* is subdivided in two subclades referring to Freetown Peninsula (FP) and remaining inland (IL) populations according to subsplit in e.g. *16S*.

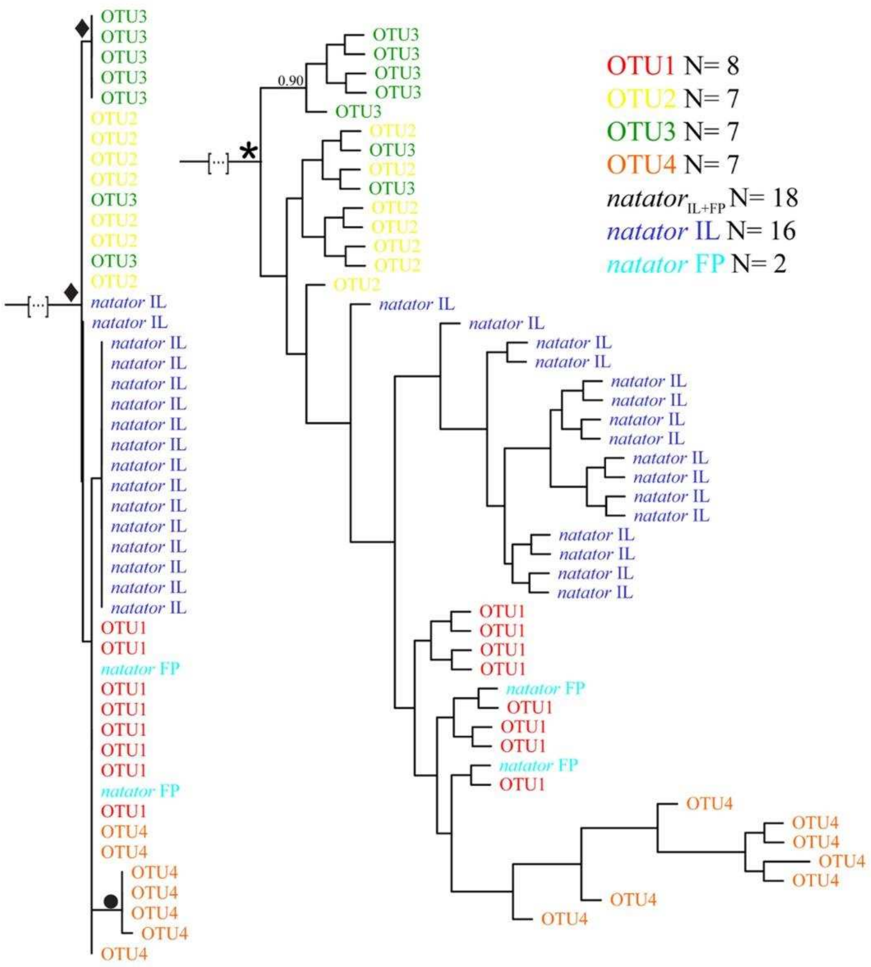

Additional file 13.5: Single gene trees of *Odontobatrachus* OTUs based on the nuclear *SIA* gene. Trees resulting from Maximum Likelihood (left) and Bayesian Inference (right), outgroups not shown. Support values are provided as Bayesian posterior probabilities (PP: * = 1.00; 0.95 ≥ ▼ ≥ 0.99; PP values below 0.70 not shown) and Boostrap support values (BS: * = 100%; 90 ≥ ♦ ≥ 99; 80 ≥ ● ≥ 89; 70 ≥ ○ ≥ 79; BS values below 50 not shown). OTU *natator* is subdivided in two subclades referring to Freetown Peninsula (FP) and remaining inland (IL) populations according to subsplit in e.g. *16S*.

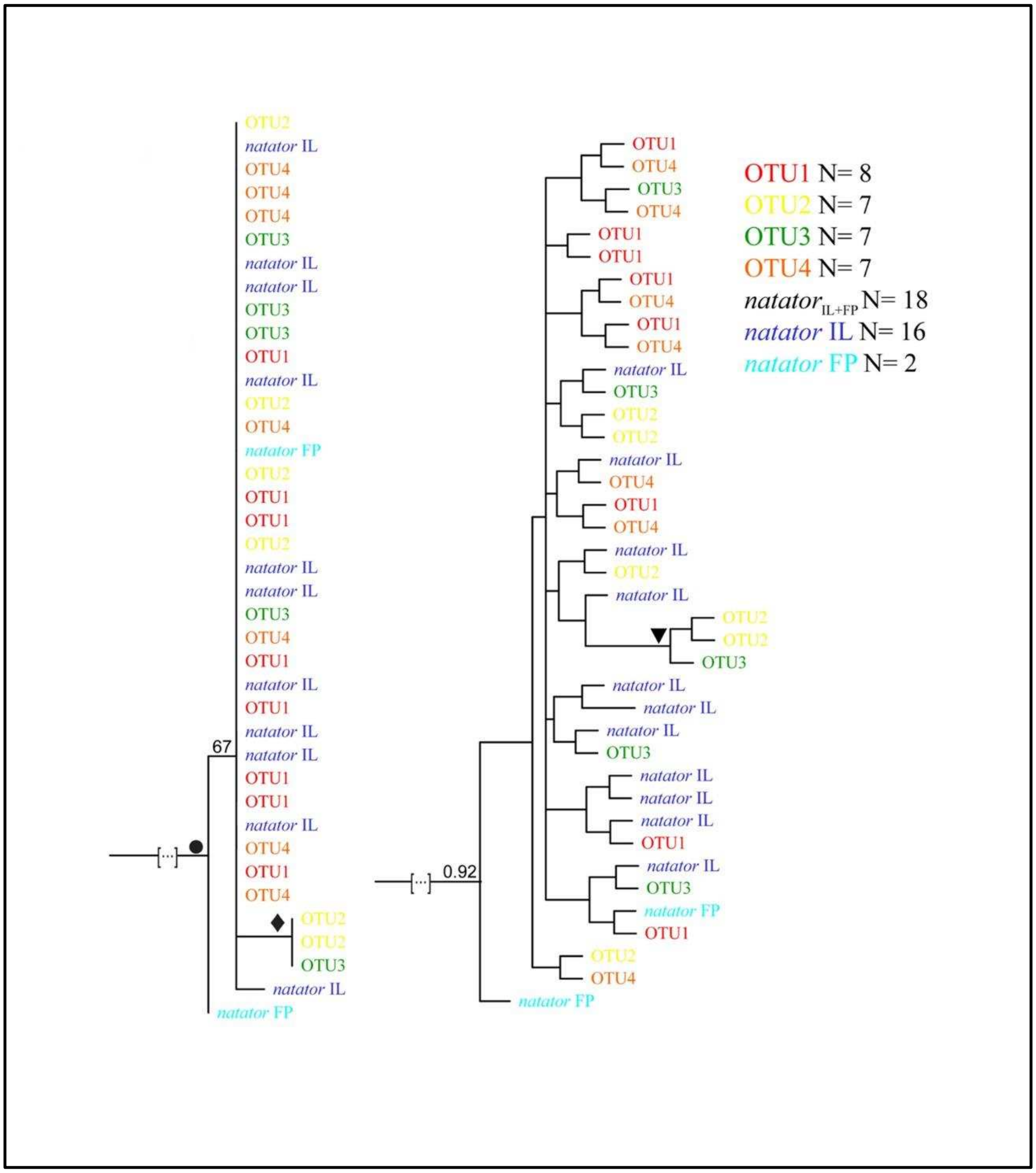

Additional file 13.6: Single gene trees of *Odontobatrachus* OTUs based on the nuclear *BDNF* gene. Trees resulting from Maximum Likelihood (left) and Bayesian Inference (right), outgroups not shown. Support values are provided as Bayesian posterior probabilities (PP: * = 1.00; 0.95 ≥ ▼ ≥ 0.99; PP values below 0.70 not shown) and Boostrap support values (BS: * = 100%; 90 ≥ ♦ ≥ 99; 80 ≥ ● ≥ 89; 70 ≥ ○ ≥ 79; BS values below 50 not shown). OTU *natator* is subdivided in two subclades referring to Freetown Peninsula (FP) and remaining inland (IL) populations according to subsplit in e.g. *16S*.
