# Supplementary material for: Multiple genetic lineages challenge the monospecific status of the West African endemic frog family Odontobatrachidae
Source: BMC Evol Biol. 2015 Apr 19;15:67. doi: 10.1186/s12862-015-0346-9 (PMC4425868; doi:10.1186/s12862-015-0346-9)

Additional file 14 (next page): Results of the jackknifing tests. Details of the jackknifing tests of variable importance to the calculated ENMs for the regularized training (left column) and testing data (middle column) and AUC values (right column) for OTUcomb, *natator* and OTU1-4 (from top to bottom). Colouration of bars as follows: without variable (pale blue), with only variable (dark blue), with all variables (red). Categories from top to bottom: **bare_4x4**: percentage of bare ground (MODIS); **glc_raw2**: vegetation derived from the near-infrared (0.78-0.89µm) wavelength of the SPOT4 satellite; **glc_raw3**: vegetation derived from the red (0.61-0.68µm) wavelength of the SPOT4 satellite; **herb_4x4**: percentage of herbaceous ground cover (MODIS); **hydro_buf_af**: distance to nearest river; **prec30_max**: highest precipitation value (wettest month); **prec30_min**: lowest precipitation value (driest month); **prec30_std**: standard deviation of the precipitation; **prec30_sum**: total annual precipitation; **srtm_c_ln_3x3**: elevational contrast calculated from the SRTM30 dataset using a 3x3 moving window; **srtm_v_ln_9x9**: elevational variance calculated from the SRTM30 dataset using a 9x9 moving window; **tmax30_max**: highest value of the maximum temperatures; **tmax30_min**: lowest value of the maximum temperatures; **tmax30_std**: standard deviation the maximum temperatures; **tmin30_max**: highest value of the minimum temperatures; **tmin30_min**: lowest value of the minimum temperatures; **tmin30_std**: standard deviation of the minimum temperatures; **tree_4x4**: percentage of woody vegetation (MODIS).

14. Results of the jackknifing tests

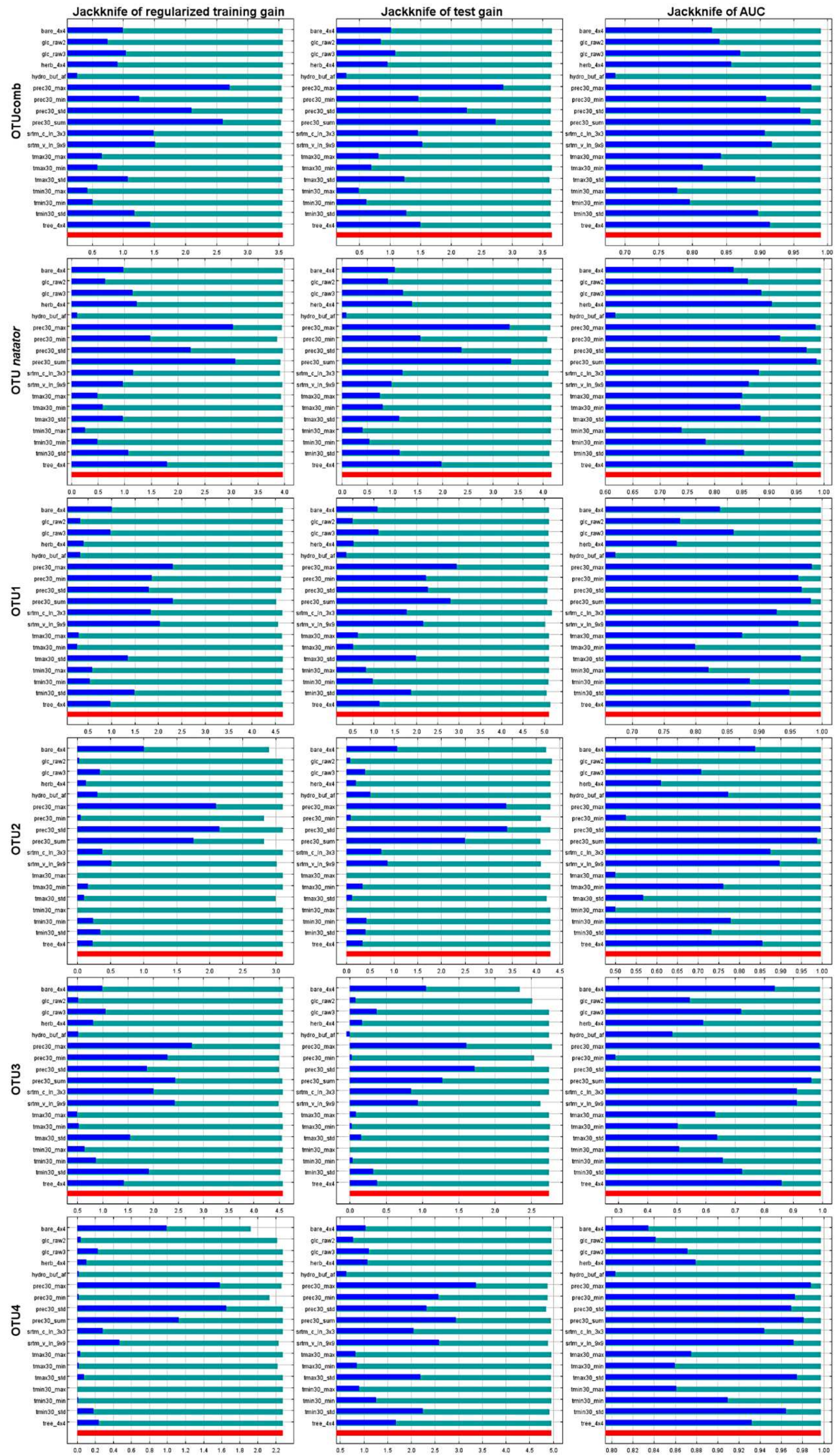

Supplement: Additional file 14: — Results of the jackknifing tests. [file 12862_2015_346_MOESM14_ESM.pdf]
